# SUMOylation Regulates TDP-43 Splicing Activity and Nucleocytoplasmic Distribution

**DOI:** 10.1007/s12035-021-02505-8

**Published:** 2021-08-14

**Authors:** AnnaMaria Maraschi, Valentina Gumina, Jessica Dragotto, Claudia Colombrita, Miguel Mompeán, Emanuele Buratti, Vincenzo Silani, Marco Feligioni, Antonia Ratti

**Affiliations:** 1 Department of Neurology, Stroke Unit and Laboratory of Neuroscience, Istituto Auxologico Italiano, IRCCS, Piazzale Brescia 20, 20149 Milan, Italy; 2 Laboratory of Neuronal Cell Signaling, EBRI Rita Levi-Montalcini Foundation, Viale Regina Elena 295, 00161 Rome, Italy; 3 “Rocasolano” Institute for Physical Chemistry, Spanish National Research Council, Serrano 119, 28006 Madrid, Spain; 4 International Centre for Genetic Engineering and Biotechnology (ICGEB), Padriciano 99, 34149 Trieste, Italy; 5 Aldo Ravelli” Center for Neurotechnology and Experimental Brain Therapeutics, Università Degli Studi Di Milano, Via A. di Rudinì 8, 20142 Milan, Italy; 6 Department of Pathophysiology and Transplantation, Dino Ferrari” Center, Università Degli Studi Di Milano, Via F. Sforza 35, 20122 Milan, Italy; 7 Department of Neurorehabilitation Sciences, Casa Di Cura del Policlinico, Via Giuseppe Dezza 48, 20144 Milan, Italy; 8 Department of Medical Biotechnology and Translational Medicine, Università Degli Studi Di Milano, Via Fratelli Cervi 93, 20090 Segrate, Milan Italy

**Keywords:** TDP-43, SUMOylation, Amyotrophic lateral sclerosis, Nucleocytoplasmic transport, Splicing

## Abstract

The nuclear RNA-binding protein TDP-43 forms abnormal cytoplasmic aggregates in the brains of amyotrophic lateral sclerosis (ALS) and frontotemporal dementia (FTD) patients and several molecular mechanisms promoting TDP-43 cytoplasmic mislocalization and aggregation have been proposed, including defects in nucleocytoplasmic transport, stress granules (SG) disassembly and post-translational modifications (PTM). SUMOylation is a PTM which regulates a variety of cellular processes and, similarly to ubiquitination, targets lysine residues. To investigate the possible regulatory effects of SUMOylation on TDP-43 activity and trafficking, we first assessed that TDP-43 is SUMO-conjugated in the nuclear compartment both covalently and non-covalently in the RRM1 domain at the predicted lysine 136 and SUMO-interacting motif (SIM, 106–110 residues), respectively. By using the SUMO-mutant TDP-43 K136R protein, we demonstrated that SUMOylation modifies TDP-43 splicing activity, specifically exon skipping, and influences its sub-cellular localization and recruitment to SG after oxidative stress. When promoting deSUMOylation by SENP1 enzyme over-expression or by treatment with the cell-permeable SENP1 peptide TS-1, the cytoplasmic localization of TDP-43 increased, depending on its SUMOylation. Moreover, deSUMOylation by TS-1 peptide favoured the formation of small cytoplasmic aggregates of the C-terminal TDP-43 fragment p35, still containing the SUMO lysine target 136, but had no effect on the already formed p25 aggregates. Our data suggest that TDP-43 can be post-translationally modified by SUMOylation which may regulate its splicing function and trafficking, indicating a novel and druggable mechanism to explore as its dysregulation may lead to TDP-43 pathological aggregation in ALS and FTD.

**Supplementary Information:**

The online version contains supplementary material available at 10.1007/s12035-021-02505-8.

## Introduction

TDP-43 is an ubiquitous RNA-binding protein (RBP) localized in the nucleus where it mainly regulates splicing but, by shuttling between the nucleus and the cytoplasm, it also controls RNA metabolism at different levels, including miRNA biogenesis, mRNA transport, stability and translation [1]. In the brain of patients suffering from the neurodegenerative diseases amyotrophic lateral sclerosis (ALS) and frontotemporal dementia (FTD), abnormal TDP-43 protein aggregates are found in the cytoplasm of both neuronal and glial cells [2–4]. In these pathological aggregates, TDP-43 is post-translationally modified by C-terminal cleavage, ubiquitination, phosphorylation and acetylation [5]. The occurring post-translational modifications (PTM) primarily seem to reduce TDP-43 solubility and to induce its aggregation [5], but acetylation was shown to decrease its RNA-binding and splicing activities [6]. However, how and whether all these PTM are interconnected in regulating TDP-43 function and in promoting its pathological aggregation is still unknown.

The PTM SUMOylation consists in the conjugation of different small ubiquitin-related modifiers (SUMO-1, 2/3 and 4) to specific lysines of the target protein through different steps which, similarly to ubiquitination, involve SUMO-E1 activating enzyme, SUMO-E2 conjugating enzyme (UBC9), and SUMO-E3 ligases. SUMOylation is reversible by means of the SENP proteases that cleave SUMO from the target proteins [7]. SUMOylation regulates a wide array of cellular processes by modifying the target protein structure, stability, solubility, localization and the interaction with protein partners [7]. The 12KDa-SUMO proteins may also interact non-covalently with the SUMO-Interaction Motif (SIM) which can be present in the target proteins [8], thus acting as a scaffold and favouring the formation of multiprotein complexes. The non-covalent SUMO conjugation is relevant for the organization and compartmentalization of sub-cellular domains, especially in the nucleus, where the PML (promyelocytic leukaemia) and the nuclear bodies as well as several spliceosome components are SUMOylated [9, 10]. SUMO conjugation to spliceosomal proteins serves for the proper assembly of the spliceosome machinery and for an efficient control of the splicing activity [11]. Within the array of cellular processes, SUMOylation regulates the nucleocytoplasmic transport (NCT) of proteins by modifying both the protein cargos to be imported/exported and the nuclear transport machinery, where RANGAP1 protein represents one of the most abundant SUMO target [12]. SUMOylation of RANGAP1 indeed promotes its translocation to the cytoplasmic side of the nuclear pore complex where it is able to establish the RanGTP/GDP gradient which is essential for NCT [13].

SUMOylation was already studied in association to neurodegenerative pathologies, such as Alzheimer’s, Parkinson’s and Huntington’s diseases, where this PTM may favour or prevent abnormal protein aggregation [14]. In ALS, SUMOylation increases mutant SOD1 protein stability and aggregation in vitro [15–18] and affects mutant VCP response to oxidative stress [19], while the RBP FUS acts as a SUMO-E3 ligase for the tumor suppressor Ebp1 and is itself SUMOylated [20].

TDP-43 was initially identified by proteomic analyses to be SUMO-2-modified in response to heat shock in HeLa cells [21] and in the insoluble fraction upon over-expression of the short splicing isoform S6 [22]. TDP-43 was also identified as a physiological SUMO-1 target in murine testis [23] and brain [24]. Recently, is has been demonstrated that the chemical inhibition of the SUMOylation pathway by anacardic acid reduces the formation of TDP-43 aggregates upon over-expression of GFP-TDP-43 in murine NSC-34 cells [24]. However, whether SUMOylation regulates TDP-43 biological activities and participates in triggering pathological TDP-43 mislocalization and aggregation is still unknown.

In this study, we therefore aimed to better characterize TDP-43 protein SUMOylation and to study its impact on TDP-43 splicing activity, subcellular distribution and aggregates formation in different experimental cell models. We also modulated SUMOylation using the deSUMOylating cell-permeable SENP1-derived peptide TS-1 or the SUMOylation inducer KCl and investigated if NCT could represent a potential druggable target in TDP-43 proteinopathies.

## Materials and Methods

### *In silico* Analyses

Four bioinformatic tools, JASSA [25], SUMO-plot (www.abcepta.com), GPS-SUMO [26, 27] and SUMO-Hydro [28], were used to predict the SUMOylation sites and the SUMO-interaction motif (SIM) of the human proteins TDP-43 (UniProtKB_Q13148-1), hnRNPA2B1 (UniProtKB_P22626-1) and NOVA1 (UniProtKB_P51513-4). The scores values obtained by GPS-SUMO analysis were manually classified as “low” (SUMOylation sites: score < 2; SIM: score < 15), “medium” (SUMOylation sites: 2 ≤ score < 3; SIM: 15 ≤ score < 30) and “high” (SUMOylation sites: score ≥ 3; SIM: score ≥ 30). Only the SUMOylation sites and SIM with a “high” score resulting from all the predictions were considered.

### Nucleotide Sequence Alignment

To analyse the phylogenetic conservation of the putative SUMOylation sites (Lys 136 and SIM3) of TDP-43, the aminoacid sequence of the RRM1 domain (106–175 aminoacids) of the human TDP-43 protein was aligned with the orthologous sequences in different species by the online pairwise sequence alignment EMBOSS Needle software (http://emboss.sourceforge.net).

### Cell Cultures, Transfection and Treatments

Human neuroblastoma SK-N-BE cells were cultured in RPMI-1640 medium (Thermo Fisher Scientific, Waltham, MA, USA) supplemented with 10% fetal bovine serum (FBS, Sigma-Aldrich, St. Louis, MO, USA), 2 mM L-glutamine, 2 g/l glucose, 1 mM sodium pyruvate, 100 U/ml penicillin and 100 μg/ml streptomycin (all from Gibco).

Human embryonic kidney (HEK) 293 T cells were maintained in DMEM medium (Thermo Fisher Scientific) supplemented with 10% FBS, 100 U/ml penicillin and 100 μg/ml streptomycin.

Cells were transiently transfected by using Lipofectamine 2000 (Thermo Fisher Scientific), following the manufacturer’s instructions. Transfections were conducted for 24 h, except for the nucleocytoplasmic distribution experiments in which cells were transfected for 48 h. *TARDBP* gene silencing was obtained by a double round transfection with 80 nM siRNA duplexes (5’-gcaaagccaagaugagccuuu-3′ and 5′-aggcucaucuuggcuuugcuu-3′) as previously described [29]. After 24-h siRNA transfection, the siRNA-resistant pFlag-CMV2-TDP-43 wild-type (WT) or K136R plasmids and the minigene constructs were transfected and cells were harvested after 48 h.

To induce stress granules formation, cells were exposed to 0.5 mM sodium arsenite (Ars) for 30 min [30]. To modulate SUMOylation, SK-N-BE cells were treated with 5 µM of the recombinant HIV Tat-linked SENP1 (TS-1) peptide for 4 h and with 60 mM KCl for 3 min.

### TS-1 Peptide

HIV Tat-linked SENP1 (TS-1) cell permeable peptide, corresponding to the 351–644 aminoacid residues of SENP1 enzyme, was obtained in *E. coli* by expressing the pTatHA-6xHis-SENP1 construct, followed by protein purification on a NTA-agarose resin. Construct cloning and protein production were previously described [31].

### Plasmids and Mutagenesis

The siRNA resistant pFlag-CMV2-TDP-43 WT, the pFlag-CMV2-TDP-43 ΔRRM1 and the pFlag-CMV2-hnRNPA2B1 constructs were previously described [32, 33]. The p3xFlag-TDP-43 WT, Q331K, M337V and A382T plasmids were kindly provided by Prof. Claudia Fallini, University of Rhode Island, Kingston, USA. The minigene pTB-*TNIKex15*, -*CFTRex9*, -*MADDex31* and the pcDNA-*STAG2ex30b* constructs for the splicing assays were obtained as previously described [29, 34, 35]. The pFlag-UBC9 construct was a kind gift of Prof. Jeremy M. Henley, University of Bristol, UK. YFP-SUMO-1 and YFP-SENP1 plasmids were previously described [36]. The pcDNA-SUMO-1 construct was generated by PCR amplification from YFP-SUMO-1 with the following primers (5’-tgggtaccaatgtctgaccaggaggcaaa-3’ and 5’-gtggatccctaaccccccgtttgttcctg-3’) and subcloned into the pcDNA3( +) plasmid (Thermo Fisher scientific). The human GFP-TDP-43, GFP-TDP-35 and GFP-TDP-25 constructs were described in [37], while the pCGN-HA-NOVA1 construct was a kind gift of Prof. Elena Battaglioli, Università degli Studi di Milano, Italy.

The SUMOylation-resistant TDP-43 (K136R) plasmid was obtained from the pFlag-CMV2-TDP-43 WT by mutagenesis of lysine 136 to arginine with the QuikChange II Site-Directed Mutagenesis Kit (Agilent Technologies), according to the manufacturer’s instruction, using the following primer pairs: 5′-gttcttatggtgcaggtcaggaaagatcttaagactggt-3′ and 5′-accagtccttaagatctttcctgacctgcaccataagaac-3′.

### Protein Extraction and Western Blot (WB) Assay

Cell pellets were resuspended in lysis buffer (20 mM Tris–HCl pH 7.5, 150 mM NaCl, 1 mM EDTA, 1 mM EGTA, 1% Triton X-100 (Sigma-Aldrich), protease/phosphatase inhibitors cocktail (Roche) and 20 nM N-ethylmaleimide (NEM) (Sigma-Aldrich) to prevent deSUMOylation [38], sonicated and incubated for 15 min on ice. BCA protein assay (Thermo Fisher Scientific) was used to quantify protein lysates and 30 μg protein samples were run on 10% NuPAGE Bis–Tris pre-cast polyacrylamide gels (Thermo Fisher Scientific) by SDS-PAGE and transferred to nitrocellulose membranes. Immunoblots were performed with specific primary antibodies (listed in Supplementary Table 1), diluted in 5% milk in TBS with 0.1% Tween-20 (Sigma-Aldrich). The HRP-conjugated secondary antibodies were detected by the Clarity ECL kit (Biorad), while the Veriblot reagent (Abcam, Cambridge, UK) was used to avoid interference of denatured IgG chains in immunoprecipation detection assays (see below). Densitometric analyses were performed using ImageJ software (NIH).

### Immunoprecipitation (IP) and Cell Fractionation

IP assays were performed using 30 μg protein G Dynabeads (Thermo Fisher Scientific) pre-coated with 2 μg of the selected antibody (Supplementary Table 1) and incubated with 200 μg of protein lysate for 45 min at room temperature (RT). Immunocomplexes were washed four times in phosphate‐buffered saline (PBS) solution 1X with 0.02% Tween-20, resolved on 10% SDS-PAGE and processed for WB analysis.

For nucleo-cytoplasm fractionation, cells were incubated in lysis buffer (PBS 1X, 0.07% Nonidet P‐40, protease/phosphatase inhibitors and 20 nM NEM). After passing lysates through a syringe needle 15 times, samples were centrifuged at 1000 × g for 30 s. An aliquot of the supernatant was removed, sonicated and analysed as whole cell lysate; the remaining supernatant and pellet (cytoplasm and nuclear fractions, respectively) were centrifuged again at 1000 × g for 30 s and the supernatant removed as cytoplasm fraction. The pellet (nuclear fraction) was resuspended in lysis buffer and sonicated. Protein concentration was determined by Bradford assay and samples (40 μg whole cell lysate and 40 μg for both cytoplasmic and nuclear protein fractions) were analyzed by WB as above.

### Molecular Dynamics (MD) Simulation

The structural ensemble consisting of the RRM domains of TDP-43 (PDB ID 4BS2) [39] was used as the initial structure for all simulations. In particular, the two RRMs with and without the bound RNA were subjected to MD runs of at least 100 ns, using both the WT and K136R sequences. All simulations were performed using GROMACS [40] with the amber99sb-ildn force field parameters [41], where the corresponding initial structures of K136R with and without bound RNA were generated using PyMOL, and placed in a cubic box filled with TIP3P water [42]. The model for non-RNA-bound RRMs were generated by removing the RNA atoms from the PDB ID 4BS2 and solvated using the same criterion. In all cases, a separation of at least 1.2 nm is left between each protein atom and the box edges, and energy-minimized using steepest descent algorithm following neutralization of charged residues with counterions to avoid artificial electrostatic repulsion. Two consecutive equilibration periods with protein atoms restrained to allow relaxation of solvent molecules were applied as follows: 1 and 5 ns under the NVT and NpT ensembles, respectively, using a modified Berendsen thermostat and the Parrinello-Rahman barostat [43, 44]. The MD simulations were run for at least 100 ns, using the LINCS algorithm [45] that affords time steps of 2 fs. These MD stages were produced under the NpT ensemble using the Nosé-Hoover [46] thermostat and Parrinello–Rahman [44] barostat to control temperature and pressure, respectively, with time constants of 0.5 and 1.0 ps. Periodic boundary conditions were applied, along with dispersion-correction to account for van der Waals interactions at distances longer than the cut-off for nonbonded interactions, which was set to 1 nm. Long-range electrostatics were calculated with the particle mesh Ewald (PME) algorithm [47].

### UV cross-linking and IP (UV-CLIP)

The intronic regions containing TDP-43 binding sites were amplified from the corresponding *TNIK, CFTR, POLDIP3, STAG2* and *MADD* minigene constructs and amplicons were cloned into the TOPO-TA vector (Thermo Fisher Scientific) downstream of the T7 promoter. UV-CLIP was performed as previously described [48]. Briefly, HindIII restriction enzyme (10U) was used to linearize 0.5 μg of each plasmid for in vitro transcription with T7 RNA polymerase and ^32^P-UTP. 200 μg protein lysates from HEK293T cells transfected with the different constructs (pFlag-CMV2-TDP-43 WT, K136R or ΔRRM1, and p3xFlag-TDP-43 WT, Q331K, M337V or A382T) to express the exogenous protein of interest were incubated with the ^32^P-radiolabeled riboprobes for UV-crosslinking. IP was then conducted on UV-cross-linked samples using the protein G Dynabeads precoated with 2 μg anti-Flag or anti-IgG antibodies (Supplementary Table 1). The immunocomplexes were washed several times in PBS 1X with 0.02% Tween-20, run on a 10% SDS-PAGE gel (Thermo Fisher Scientific) and analysed by autoradiography.

### Splicing Assays

Total RNA (1.5 μg) was isolated using TriZol reagent (Thermo Fisher Scientific) treated with 1U DNaseI (Roche) for 20 min at 37 °C and then retro-transcribed using 1U SuperScript II Reverse Transcriptase (Thermo Fisher Scientific) and 3 μM oligo-dT. RT-PCR was performed using 300 nM specific primer pairs for the minigene constructs or the endogenous gene targets (Supplementary Table 2) for 26–35 cycles. *GAPDH* gene was used for sample normalization. Amplicons were loaded on 2% agarose gels and quantified by densitometric analyses using ImageJ software (NIH). The alternative splicing events of interest (exon skipping or inclusion) were represented as percentage of the total splicing isoforms. Transfection efficiency of HEK293T cells with the pFlag-CMV2-TDP-43 WT or K136R plasmids was assessed by WB for all the tested conditions in parallel to RT-PCR assays.

### Immunofluorescence (IF)

Cells were fixed with 4% paraformaldehyde in PBS 1X for 20 min at RT, treated with cold methanol for 3 min and permeabilized with 0.3% Triton X-100 for 5 min. The blocking solution (10% normal goat serum (NGS, Gibco) in PBS 1X) was used for 20 min at RT before the incubation with primary antibodies (Supplementary Table 1) performed in blocking solution for 2 h at 37 °C. The fluorescent-tagged secondary antibodies Alexa Fluor 488 and 555 (Thermo Fisher Scientific) were used for detection. Nuclei were visualized by DAPI staining (Roche, Basilea, Switzerland). Coverslips were mounted onto glass slides using FluorSave mounting medium (Merck, Darmstadt, Germany). Images were acquired as Z-stacks (0.2 μm step size) at 60 × magnification using confocal inverted microscope (Nikon Eclipse C1, Minato, Japan) or the epifluorescence microscope (Nikon).

### Image Analyses

The sub-cellular distribution of the protein of interest was evaluated by counting at least 80 cells per condition. Different groups were defined according to the sub-cellular localization (nuclear, cytoplasmic, nuclear + cytoplasmic) or distribution (puncta, aggregates) of the analysed protein and data presented as percentage of cells counted in at least three independent experiments. The size (area) of GFP-TDP-25 aggregates was measured by ImageJ software according to four arbitrarily assigned groups (< 0.2 µm^2^; [0.2–0.5[ µm^2^; [0.5–1 µm^2^]; > 1 µm^2^).

### Statistical Analyses

Statistical analyses were conducted with GraphPad PRISM 5 software package. Before applying the appropriate parametric or non-parametric tests, data normality was evaluated by the D'Agostino-Pearson Omibus test. One-/Two-way ANOVA and Chi-Square followed by appropriate post-hoc tests were applied to compare multiple groups. Data were presented as mean ± s.e.m. (standard error of mean) or s.d. (standard deviation) of at least three independent experiments. Significance value was defined as *p < 0.05, **p < 0.01, ***p < 0.001.

The summary statistics of all the experimental data and figures is shown in Supplementary Table 3.

## Results

### *In Silico *and* In Vitro Analyses of TDP-43 SUMOylation*

**Fig. 1:**
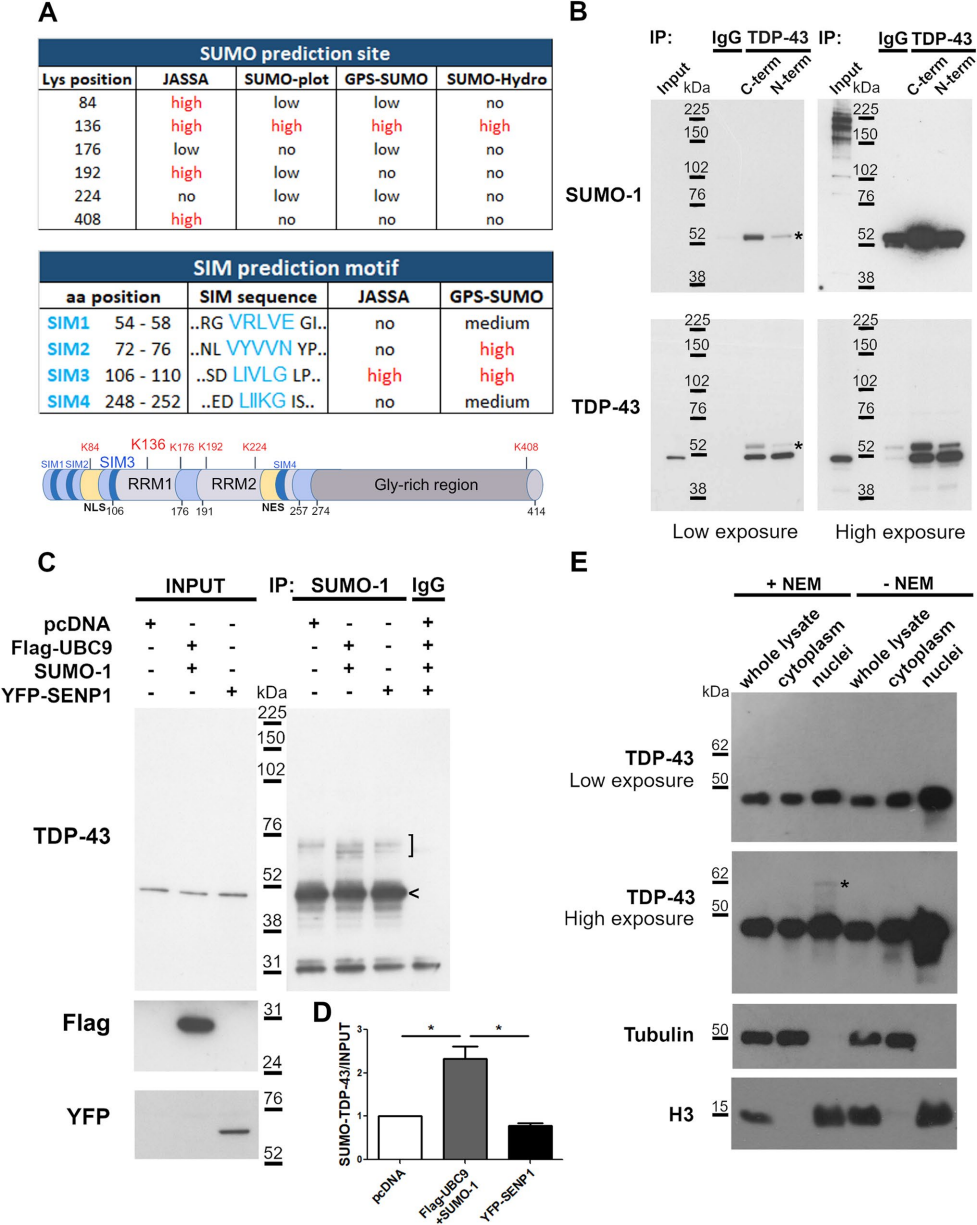
Characterization of TDP-43 protein SUMOylation. (**a**) In silico prediction analysis of TDP-43 SUMOylation sites (*top table*) and SIM motifs (*bottom table*) performed by different bioinformatic tools as indicated (the “high” score is indicated in red). Schematic representation of TDP-43 protein with the putative lysine (K136) and SIM motif (SIM3) predicted with a “high” score by all programs is shown. (**b**) Representative WB images of immunoprecipitation (IP) assay on SK-N-BE cell lysates with NEM reagent performed with two anti-TDP-43 antibodies recognizing the N-term or C-term TDP-43 domain and immunoblotted for SUMO-1 and TDP-43. IgG was used as negative control for IP (*n* = 3 independent experiments); *asterisk,* SUMOylated TDP-43 protein. (**c**) WB images showing IP assay on SK-N-BE cell lysate transfected with Flag-UBC9 and SUMO-1 or with YFP-SENP1 constructs. IP was performed using the anti-SUMO-1 and IgG (negative control) antibodies (*n* = 3 independent experiments; *bracket*, SUMOylated forms of TDP-43 protein; *arrowhead*, recovered TDP-43 protein non-covalently bounded to SUMO-1. (**d**) Image quantification of WB data presented in (**c**) (mean ± s.d; One-way ANOVA and Tukey post hoc test; *n* = 3; *p <  0.05). (**e**) Representative WB images of nucleo-cytoplasm fractionation of SK-N-BE cell lysates with or without NEM (*n* = 5 independent experiments; *asterisk*, the SUMOylated TDP-43 protein)

Analysis of TDP-43 SUMOylation was first assessed in silico by using four different bioinformatic tools (JASSA, SUMO-plot, GPS-SUMO and SUMO-Hydro) which consistently predicted the Lys 136 residue and a hydrophobic SUMO-interacting motif (SIM3, 106–110 residues) as SUMOylation sites, suggesting both a Lys-mediated covalent and a SIM-mediated non-covalent binding of SUMO proteins to TDP-43 (Fig. 1a). The two predicted SUMO binding sites are both located within the TDP-43 RRM1 domain, which is highly conserved along phylogenesis (Supplementary Fig. 1a) and has an important function for target RNA recognition and binding.

Based on the in silico analyses, the SUMOylation state of the endogenous TDP-43 protein was then assessed in vitro by immunoprecipitation (IP) of human neuroblastoma SK-N-BE cell lysates with two different antibodies recognizing aminoacidic sequences at the N-terminal or at the C-terminal region of TDP-43. We found that a fraction of the endogenous TDP-43 protein was physiologically SUMOylated and that the SUMOylated form was more efficiently recovered using the C-terminal antibody, while the unmodified TDP-43 was similarly immunoprecipitated by both antibodies (Fig. 1b). Similar results were obtained in HEK293T cells in which the SUMO-modified TDP-43 was preferentially recovered by the C-terminal antibody although both TDP-43 antibodies were able to immunoprecipitate the unmodified protein (Supplementary Fig. 1b). These findings, together with the in silico analyses, suggest that a fraction of endogenous TDP-43 is likely to be SUMOylated at the N-terminal domain which is supposed to become less recognizable by the N-terminal antibody when modified by SUMO binding (Fig. 1a).

We over-expressed SUMO-1 and UBC9 plasmids together or SENP1 construct in SK-N-BE cells to induce SUMOylation or de-SUMOylation, respectively. By IP assay with SUMO-1 antibody, we observed that the SUMOylated TDP-43 form increased in condition of SUMO-1/UBC9 over-expression by 2.3 folds, while SENP1 over-expression did not induce changes compared to control cells (Fig. 1c,d). Moreover, the SUMO-1 antibody was able to recover TDP-43 also at its native molecular weight (Fig. 1c), suggesting that TDP-43/SUMO-1 interaction may occur also through a non-covalent binding, consistent with the presence of the SIM3 region predicted in silico (Fig. 1a). In line with these results, an increase of the covalently SUMO-modified TDP-43 was observed also in HEK293T cells upon SUMO-1 over-expression, while the unmodified TDP-43 form was similarly recovered by SUMO-1 antibody both in physiological condition and after induction of SUMOylation, confirming the non-covalent SUMO-1 binding to TDP-43 also in non-neuronal cells (Supplementary Fig. 1c).

By subcellular fractionation assays, we further assessed that the SUMO-modified TDP-43 protein was totally localized in the nucleus as shown by the specific band that disappeared when the N-Ethylmaleimide (NEM) reagent, used to inhibit protein de-SUMOylation, was omitted in the lysis buffer (Fig. 1e).

### Characterization of the SUMOylation-resistant TDP-43 Protein

**Fig. 2:**
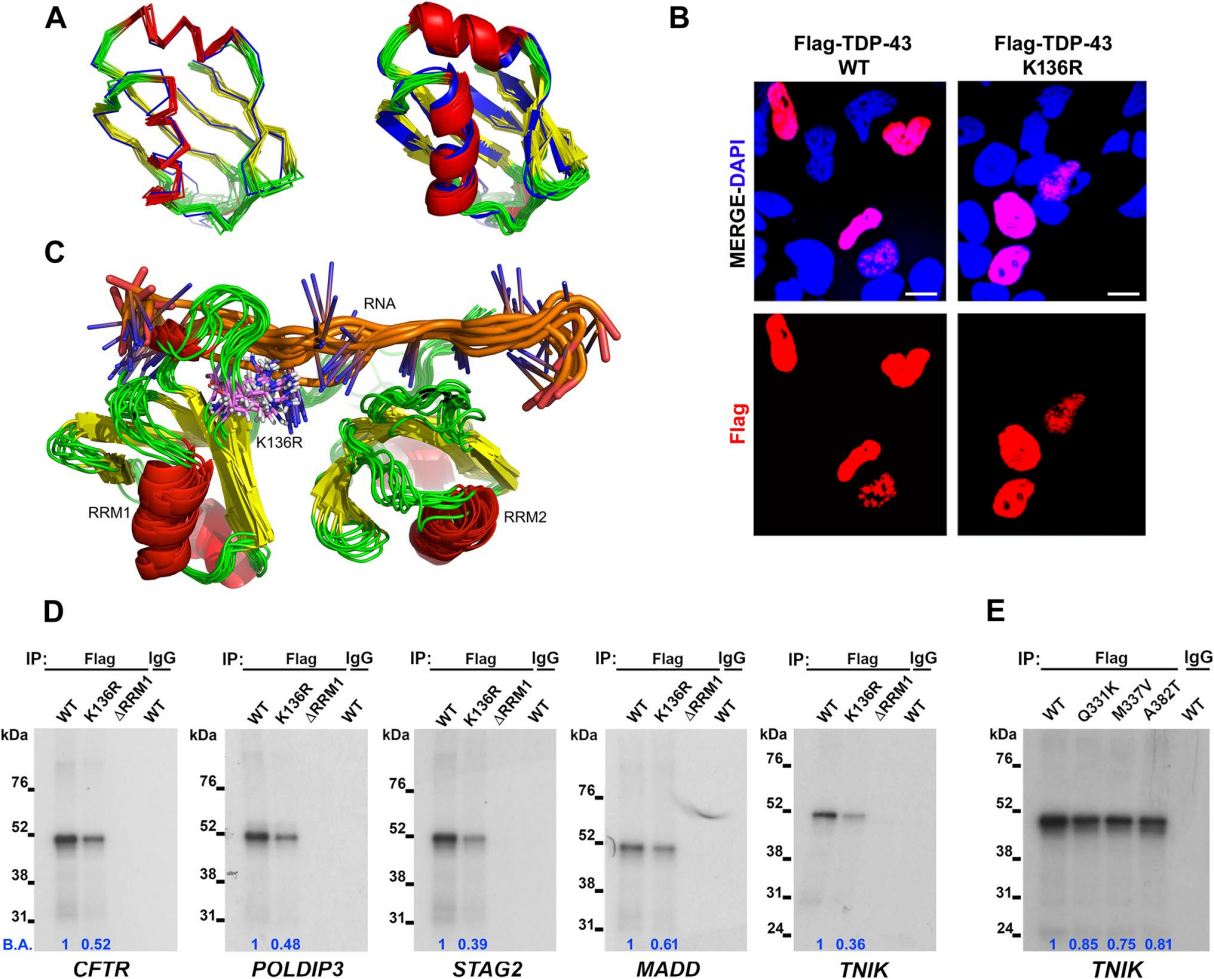
Characterization of the SUMO-resistant TDP-43 K136R protein. (**a**) Superimposition of ten TDP-43 K136R structures extracted every 10 ns from a 100-ns MD trajectory (strands in yellow, helices in red, unstructured segments and loops in green) onto the lowest-energy conformer from the wild-type NMR ensemble 4BS2 (all elements in blue). Ribbon and cartoon representations are shown (left and right images, respectively). Superimposition of the structures reflects that K136R does not distort the fold of the RRM1, which is evinced by an averaged RMSD value over all ten frames for N, CA, C', and O backbone atoms as low as 1.17 Å. (**b**) IF images showing the subcellular distribution of the Flag-tagged TDP-43 WT and K136R proteins (red) in SK-N-BE cells. DAPI (blue) was used for nuclear staining. Scale bar, 10 µm. (**c**) Simulated ensemble (10 structures extracted every 10 ns from a 100-ns MD run) on the TDP-43 K136R variant. Protein-RNA interactions are preserved for the 136 site when Lys is replaced by Arg. The RRMs are coloured in red (helices), yellow (strands) and green (loops and disordered segments). The RNA molecules are shown in blue (bases) and orange (backbone). R136 is depicted in violet to highlight the K136R substitution. (**d**) SDS-PAGE of UV-CLIP experiments using Flag-TDP-43 WT, K136R or ΔRRM1 recombinant proteins from transfected HEK293T cell lysates and the ^32^P-radiolabelled *CFTR, POLDIP3, STAG2*, *MADD* and *TNIK* riboprobes. Anti-Flag antibody was used for IP and the anti-IgG antibody was used as a negative control. (**e**) UV-CLIP assay performed on HEK293T protein lysates containing recombinant Flag-tagged TDP-43 WT, Q331K, M337V and A382T proteins by using the anti-Flag antibody for IP and the *TNIK* radiolabelled riboprobe. IgG was used as negative control in IP. The RNA-binding affinity (B.A.) of the different recombinant proteins was calculated versus the TDP-43 WT protein by densitometric analysis

Our in silico analyses and in vitro experiments suggested that TDP-43 protein is SUMO-1-modified, likely in the RRM1 domain, both covalently and non-covalently in neuronal-like and non-neuronal cell lines. To better study the role of covalent SUMO-1 binding to TDP-43, we generated a SUMOylation-resistant TDP-43 protein in which the putative Lys 136 was mutated to Arg (K136R). The K136R substitution is expected to have a negligible impact on the structure of the RRM1 domain, considering the similarity between Arg and Lys and the observation that Lys 136 is Arg in *C. elegans*. This was ascertained using Molecular Dynamics (MD) simulations, which showed an average root mean square deviation (RMSD) of 1.17 Å over the course of a 100-ns MD run for the backbone atoms of the full RRM1 in K136R with respect to the WT system. This low value indicated that the K136R substitution did not disrupt the structural integrity of the RRM1 domain (Fig. 2a). After computationally excluding a major impact of the K136R substitution on TDP-43 protein structure, we investigated the sub-cellular localization of the SUMOylation-resistant TDP-43 in SK-N-BE cells by immunofluorescence (IF) analysis and we observed that it was mainly localized in the nucleus, similarly to the wild-type protein (Fig. 2b).

Next, we explored the RNA-binding properties of K136R, as the NMR structural ensemble and available X-ray crystallographic structures of RNA-bound TDP-43 RRMs showed that K136 itself establishes a number of important contacts with the target RNA and is therefore an important site for RNA-RRM1 interaction [39, 49, 50]. Indeed, the analyses of the 20 structures in the NMR ensemble showed that the side chain of K136 is not tied down to a buried conformation, but rather exposed and available for interacting with RNA (Supplementary Fig. 2) or other possible substrates, as investigated in the present study. MD simulations showed that, in the SUMO-resistant variant K136R, the bulkier Arg residue is still flexible, exposed and able to maintain a number of direct contacts with the target RNA (Fig. 2c). To experimentally corroborate that the RNA-binding capability of the mutant TDP-43 K136R protein is broadly maintained, we performed UV-crosslinking immunoprecipitation (UV-CLIP) assays to test the binding activity of the SUMOylation-resistant TDP-43 protein to its RNA targets. To this purpose, we selected five RNA splicing targets containing both the canonical TDP-43 consensus binding sequence UGn (*CFTR*, *MADD* and *TNIK* genes) and the non-canonical one (*POLDIP3* and *STAG2* genes). Our UV-CLIP results showed that the mutant TDP-43 K136R bound to all RNA targets, although with a lower binding affinity compared to the wild-type protein (Fig. 2d), in a condition where both exogenous proteins were expressed similarly in the lysates used in the assay (Supplementary Fig. 3). Importantly, the TDP-43 ΔRRM1 protein, which is deleted of the RRM1 and was used as negative control in the assay, showed the total inability of binding to all the RNA targets analysed (Fig. 2d). Finally, also the recombinant TDP-43 proteins carrying the ALS-associated mutations Q331K, M337V and A382T in the C-terminal domain were used to test their RNA-binding activity to the selected RNA targets. All these mutant TDP-43 proteins also showed a slight decrease of their RNA binding capacity compared to the wild-type protein (Fig. 2e). Taken together, these observations indicate that the K136 residue is well exposed and possibly accessible for SUMOylation and that the SUMO-resistant K136R variant did not affect the folding of TDP-43 RRMs. Although showing a somewhat lower binding affinity, the K136R substitution did not disrupt the binding of target RNAs.

### Analysis of the Splicing Activity of the SUMOylation-resistant TDP-43 Protein

**Fig. 3:**
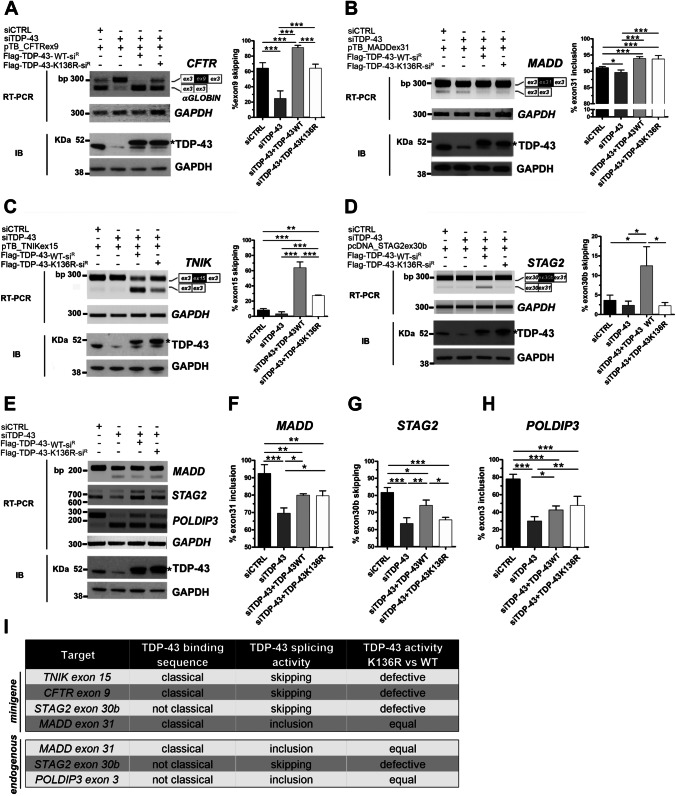
Splicing activity of the SUMO-resistant TDP-43 K136R protein. (**a–d**) Representative RT-PCR (*Upper panels*) and immunoblot (IB) (*Lower panels*) images of minigene splicing assays in HEK293T cells knocked-down for *TARDBP* gene and co-transfected with the siRNA resistant Flag-TDP-43 WT or K136R constructs and the pTB_minigenes *CFTRex9* (**a**), *MADDex31* (**b**), *TNIKex15* (**c**) or the pcDNA_minigene *STAG2* (**d**) as indicated. *GAPDH* was used for data normalization in both RT-PCR and IB assays. *Asterisk*, exogenous Flag-tagged TDP-43 WT or K136R proteins. Densitometric analyses of *CFTRex9* skipping (**a**), *MADDex31* inclusion (**b**), *TNIKex15* skipping (**c**) and *STAG2ex30b* skipping (**d**) data from the minigene assays (mean ± s.d.; One-way ANOVA and Tukey post hoc test; *n* = at least 3 independent experiments; *p <  0.05; **p <  0.01; ***p <  0.001). (**e**) Representative RT-PCR (*Upper panels*) and IB images (*Lower panels*) of endogenous *MADD*, *STAG2* and *POLDIP3* alternative splicing in HEK293T cells, knocked-down for *TARDBP* and transfected with the siRNA-resistant Flag-TDP-43 WT or K136R constructs. *GAPDH* was used for sample normalization. (**f**–**h**) Densitometric analyses of endogenous *MADDex31*, *STAG2ex30b* and *POLDIP3ex3* splicing (mean ± s.d.; One-way ANOVA and Tukey post hoc test; *n* = at least 3 independent experiments; *p <  0.05; **p <  0.01; ***p <  0.001). (**i**) Summary table of the splicing activity of TDP-43 K136R versus the WT protein on minigenes and endogenous gene targets. The types of consensus binding sequence and splicing event for the analysed target are also reported for comparison

We then investigated if the reduced RNA-binding capacity of the SUMOylation-resistant TDP-43 protein could compromise its splicing activity by performing minigene splicing assays in HEK293T cells knocked-down for TDP-43. By over-expressing siRNA-resistant wild-type or K136R TDP-43 constructs, we compared their ability to rescue splicing defects induced by endogenous *TARDBP* gene silencing. We used minigene plasmids expressing the splicing targets already tested in the UV-CLIP assays and including the target exons and the flanking intronic regions for *CFTR* (exon 9), *MADD* (exon 31), *TNIK* (exon 15) and *STAG2* (exon 30b) (Fig. 3a–d). Upon *TARBDP* gene silencing, the skipping of *CFTR* exon 9 (20.6%) and the inclusion of *MADD* exon 31 (89.7%) significantly decreased in comparison to siRNA-control condition (62.2% and 91.1%, respectively) (Fig. 3a,b), as previously described [35, 51]. Although not statistically significant, the skipping of *TNIK* exon 15 (3.2%) and *STAG2* exon 30b (2.3%) was also decreased compared to control cells (8.4% and 3.7% respectively) (Fig. 3c,d) as already demonstrated [29, 35]. When we over-expressed the siRNA-resistant wild-type TDP-43 protein, all the analysed splicing events were rescued with a splicing activity that, in line with an increased amount of TDP-43 production following transfection, was significantly higher (*CFTR*: 90.3%; *MADD*: 93.9%; *TNIK*: 64%; *STAG2*: 12.5%) with respect to the siRNA-control condition (Fig. 3a–d). Upon TDP-43 K136R over-expression, *MADD* exon 31 inclusion was rescued similarly to the exogenous wild-type TDP-43 protein (93.8% and 93.9%, respectively; Fig. 3b). In contrast, the mutant TDP-43 K136R promoted the skipping of *CFTR* exon 9 (62.8%) and *TNIK* exon 15 (27.5%) to a lower extent compared to the wild-type protein (Fig. 3a,c), but was not able to promote *STAG2* exon 30b skipping (2.3%) (Fig. 3d).

By further extending our splicing analysis to endogenous TDP-43 splicing targets, including *MADD* and *STAG2* genes, we observed that, upon *TARDBP* gene silencing, TDP-43 K136R was as efficient as the recombinant wild-type protein in rescuing exon inclusion activity of *MADD* exon 31 (79.6% *vs* 79.9%), while it was less effective in the skipping activity on *STAG2* exon 30b (65.7% *vs* 74.1%) (Fig. 3e–g), confirming the results obtained in the minigene splicing assays (Fig. 3c–d). To investigate if these results might depend specifically on the type of alternative splicing event regulated by TDP-43 (exon skipping *vs* exon inclusion), we also studied the well-known splicing target *POLDIP3*, containing a non-canonical recognition motif and whose exon 3 inclusion is promoted by TDP-43 [52, 53]. We observed that *POLDIP3* exon 3 inclusion decreased to 29.8% after *TARDB*P gene silencing compared to the control condition (78%), as expected, but was rescued similarly by both the wild-type (42.5%) and the K136R (47.7%) TDP-43 proteins (Fig. 3e,h).

Altogether, our results show that the TDP-43 K136R protein has a less effective exon skipping activity, but retains an exon inclusion activity comparable to the wild-type protein in regulating target gene splicing. Moreover, this differential splicing activity occurs independently on the type of the consensus binding sequence present in the target intronic region (classical UGn sequence *vs* not classical recognition motif) (Fig. 3i).

### TDP-43 SUMOylation and Stress Granules Formation

**Fig. 4:**
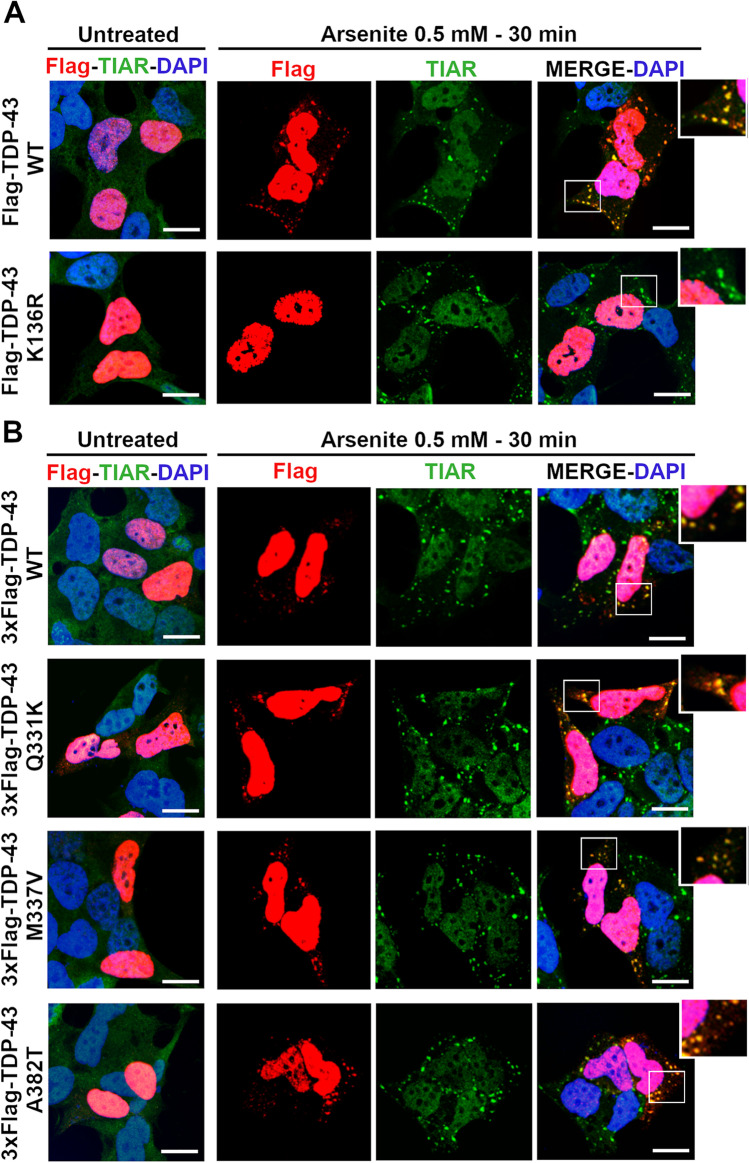
The SUMO-resistant TDP-43 and stress granules formation. Representative IF images of SK-N-BE cells transfected with (**a**) Flag-TDP-43 WT or K136R and (**b**) the ALS-associated TDP-43 mutant Q331K, M337V and A382T constructs and treated with sodium Arsenite (0.5 mM, 30 min). In the untreated condition, the SG marker TIAR (green) and Flag (red) staining are shown in the merged images. Nuclei are visualized by DAPI (blue) in all the merged panels. Scale bar, 10 µm

Besides acting mainly as a splicing factor, TDP-43 is also implicated in cell response to stress and in stress granules (SG) formation in the cytoplasm [54]. We previously showed that TDP-43 is able to form SG if its RNA-binding ability is preserved (Colombrita et al., 2009). Given the observed decreased binding affinity of the SUMO-mutant TDP-43 protein to its target RNAs (Fig. 2d), we investigated its ability to be recruited into SG upon an acute oxidative stress stimulus. By analysing the sub-cellular distribution of TDP-43 in human neuroblastoma SK-N-BE cells exposed to sodium arsenite (0.5 mM) for 30 min, we observed that the TDP-43 K136R protein remained in the nucleus, while the wild-type TDP-43 formed cytoplasmic foci co-localizing with the SG marker TIAR, as expected (Fig. 4a). When we analysed the mutant ALS-associated TDP-43 proteins (Q331K, M337V and A382T), we observed that they were all recruited into SG as the wild-type TDP-43 upon arsenite insult (Fig. 4b).

### TDP-43 Nucleocytoplasmic Trafficking After Modulation of SUMOylation

**Fig. 5:**
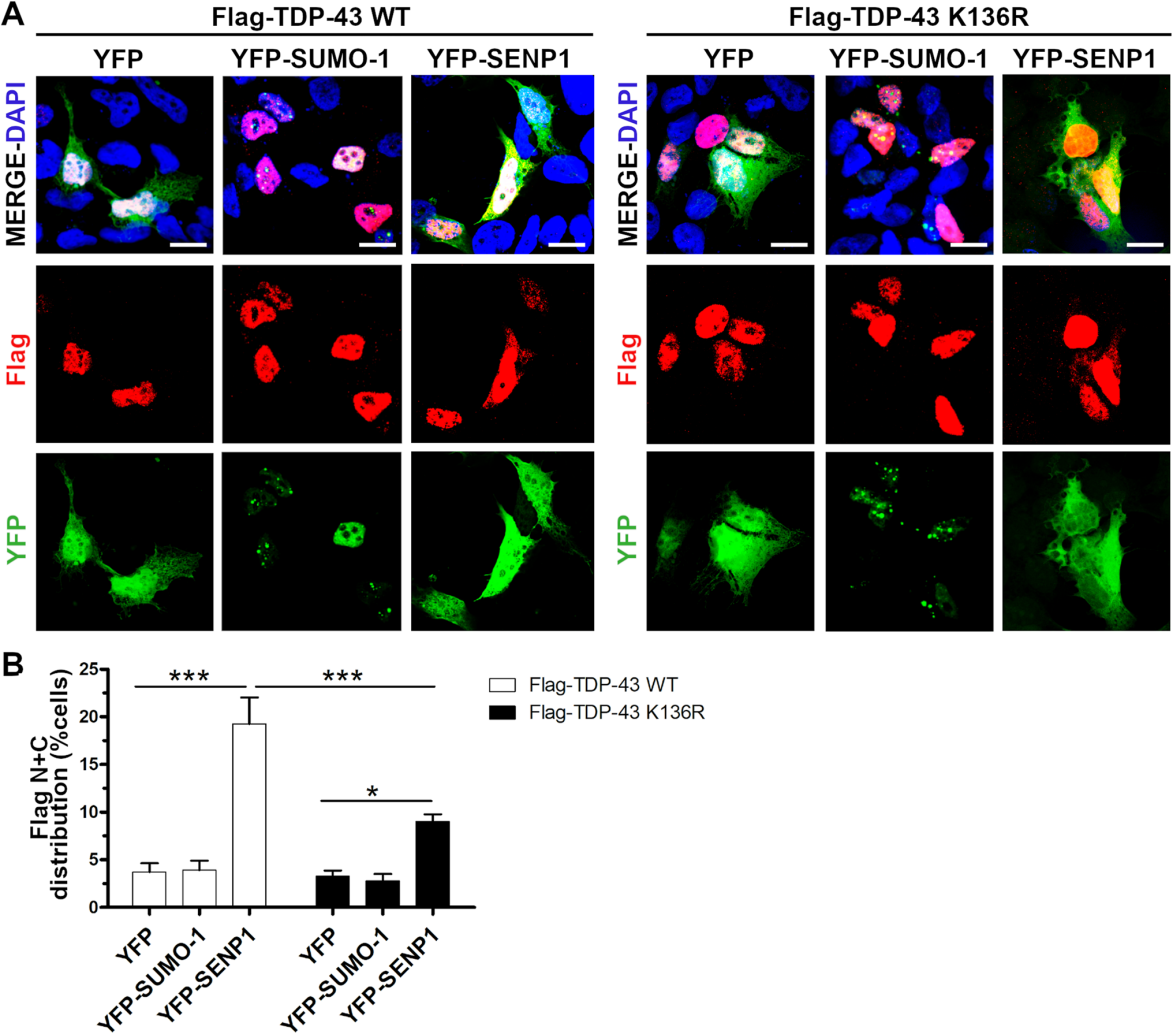
Sub-cellular distribution of TDP-43 upon modulation of SUMOylation. (**a**) IF images of SK-N-BE cells co-transfected with Flag-TDP-43 WT or K136R and YFP-SUMO-1 or YFP-SENP1 as indicated. Nuclei were stained with DAPI shown in the merged images. Scale bar, 10 µm. (**b**) Statistical analyses of the nuclear and cytoplasmic (*N* + *C*) distribution of the Flag-TDP-43 WT and K136R proteins in YFP-SUMO-1 or YFP-SENP1 expressing cells (mean ± s.e.m.; Two-way ANOVA and Bonferroni post-test; *n* = 3 independent experiments; *p <  0.05; ***p <  0.001)

To investigate the role of SUMOylation in the nucleocytoplasmic trafficking of TDP-43, we analysed the sub-cellular localization of the wild-type and the SUMOylation-resistant TDP-43 proteins upon modulation of SUMOylation by SUMO-1 or SENP1 over-expression in SK-N-BE cells. Both exogenous TDP-43 proteins distributed mainly in the nucleus in the mock-transfected cells, but 3.7% of TDP-43 WT-positive and 3.3% of TDP-43 K136R-positive cells showed both a nuclear and a diffused localization in the cytoplasm (Fig. 5a,b). After induction of SUMOylation by YFP-SUMO1 over-expression, we observed no differences in the sub-cellular distribution of the two TDP-43 proteins compared to the control condition (3.9% TDP-43 WT-transfected and 2.8% TDP-43 K136R-transfected cells with TDP-43 protein also localized in the cytoplasm) (Fig. 5a,b). On the other hand, when we induced de-SUMOylation by over-expressing YFP-SENP1 construct, we observed a significant increase of cells showing also a cytoplasmic distribution of the WT (19.3%) and K136R (9%) TDP-43 proteins, although the number of cells with the SUMOylation-resistant TDP-43 mislocalized in the cytoplasm was significantly lower compared to TDP-43 WT-expressing cells (Fig. 5b). We never observed a complete cytoplasmic mislocalization of the two exogenous proteins associated to their nuclear depletion after YFP-SENP1 over-expression.

Since our results showed that TDP-43 mislocalization in the cytoplasm increased upon promoting de-SUMOylation and it occurred at a lower extent for the SUMOylation-resistant TDP-43 protein, we then evaluated if such effect might depend on the modulation of the NCT system or was specific for TDP-43. We considered two splicing factors, the TDP-43 interactor hnRNPA2B1 and NOVA1, which, like TDP-43, mainly localize in the nucleus, but also shuttle between the nucleus and the cytoplasm [55, 56]. In silico analysis did not predict any SUMOylation site nor SIM for hnRNPA2B1, while NOVA1 was highly predicted to have Lys 219 as a putative SUMO-binding site by two out of the three programs used (Supplementary Fig. 4a,b). By IF assays, we observed that, in control condition, recombinant NOVA1 prevalently localized in the nucleus with 16.4% of transfected cells showing both a nuclear and a cytoplasmic distribution of the protein (Supplementary Fig. 4c,e). This percentage decreased, although not significantly, to 7.5% upon stimulation of SUMOylation by YFP-SUMO-1 over-expression, while it showed a trend to increase (19.9%) when YFP-SENP1 was over-expressed (Supplementary Fig. 4e). Conversely, the sub-cellular localization of hnRNPA2B1 was not affected by either YFP-SUMO-1 or YFP-SENP1 over-expression and remained entirely nuclear in all the experimental conditions (Supplementary Fig. 4d,f).

Our data indicate that the nucleocytoplasmic trafficking of TDP-43 and NOVA1 splicing factors is not regulated exclusively by the SUMO-modulation of the NCT system, but also depends on their intrinsic property of being SUMOylable targets.

### TDP-43 Cytoplasmic Mislocalization After Treatment with the deSUMOylating TS-1 Peptide

**Fig. 6:**
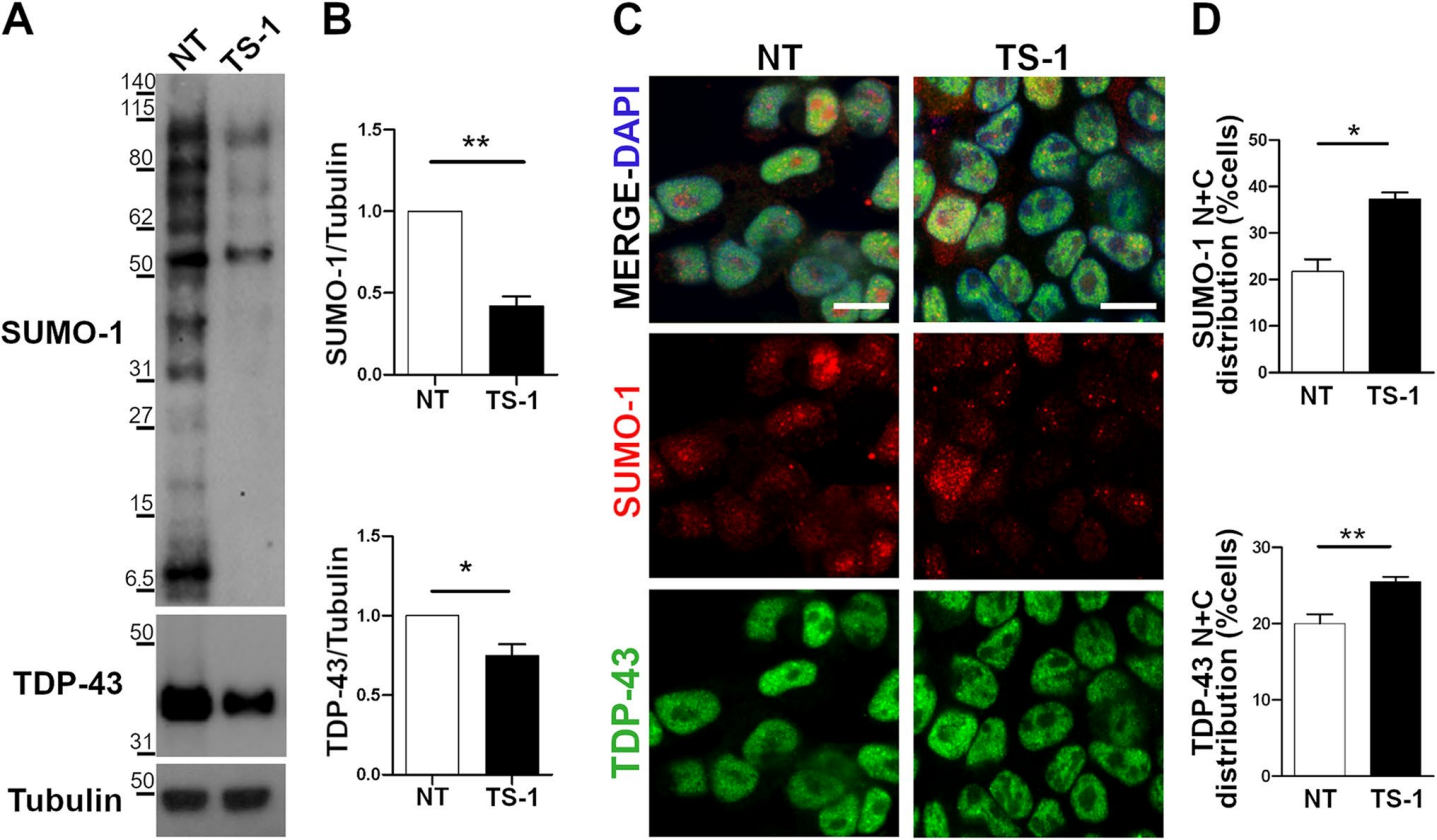
TDP-43 nucleocytoplasmic distribution upon treatment with the cell-permeable TS-1 peptide. (**a**–**b**) Representative WB images and densitometric analyses of total SUMO-1 and TDP-43 protein levels before (NT) and after TS-1 treatment (5 µM, 4 h) in SK-N-BE cells. α-Tubulin was used for data normalization (mean ± s.e.m; *n* = 5 independent experiments; Unpaired t-test; *p <  0.05; **p <  0.01). (**c**) IF images of endogenous TDP-43 (green) and SUMO-1 (red) proteins in untreated (NT) and TS-1-treated SK-N-BE cells. DAPI was used for nuclei staining. Scale bar, 10 µm. (**d**) Statistical analyses of the nuclear and cytoplasmic (N + C) distribution of the endogenous SUMO-1 and TDP-43 proteins in TS-1-treated and control (NT) conditions (mean ± s.e.m.; *n* = 4 independent experiments; at least 100 cells analysed/condition; Unpaired t-test; *p <  0.05; **p <  0.01)

In order to confirm the effect of de-SUMOylation on TDP-43 molecular trafficking in physiological conditions, we treated SK-N-BE cells with the cell-permeable peptide TS-1 that we previously generated from the C-terminal domain of the SENP1 enzyme and proved to promote protein de-SUMOylation [31], and analysed the sub-cellular distribution of the endogenous TDP-43 protein. By western blot analysis, we first confirmed that 5 µM TS-1 applied for 4 h was able to promote de-SUMOylation by inducing a significant decrease (0.42X) of the total amount of SUMOylated proteins compared to untreated cells and we found that TS-1 significantly decreased also TDP-43 protein content (0.75X) (Fig. 6a,b). IF analysis revealed that TS-1 treatment caused changes also in SUMO-1 sub-cellular distribution because the proportion of cells showing both a nuclear and a cytoplasmic localization significantly increased from 21.75% to 37.25% in TS-1-treated cells (Fig. 6c,d).

When we quantified TDP-43 sub-cellular distribution, the majority of SK-N-BE cells showed a nuclear localization of the protein before and after TS-1-treatment, with no cells showing an exclusive TDP-43 cytoplasmic localization, but the percentage of cells showing a nuclear and cytoplasmic TDP-43 localization significantly increased from 20% in untreated to 25.5% in TS-1-treated cells (Fig. 6c–d), indicating that TDP-43 trafficking can be modulated by the de-SUMOylating cell-permeable TS-1 peptide.

### Induction of SUMOylation by KCl and Analysis of its Effect on TDP-43 Nucleocytoplasmic Localization

**Fig. 7:**
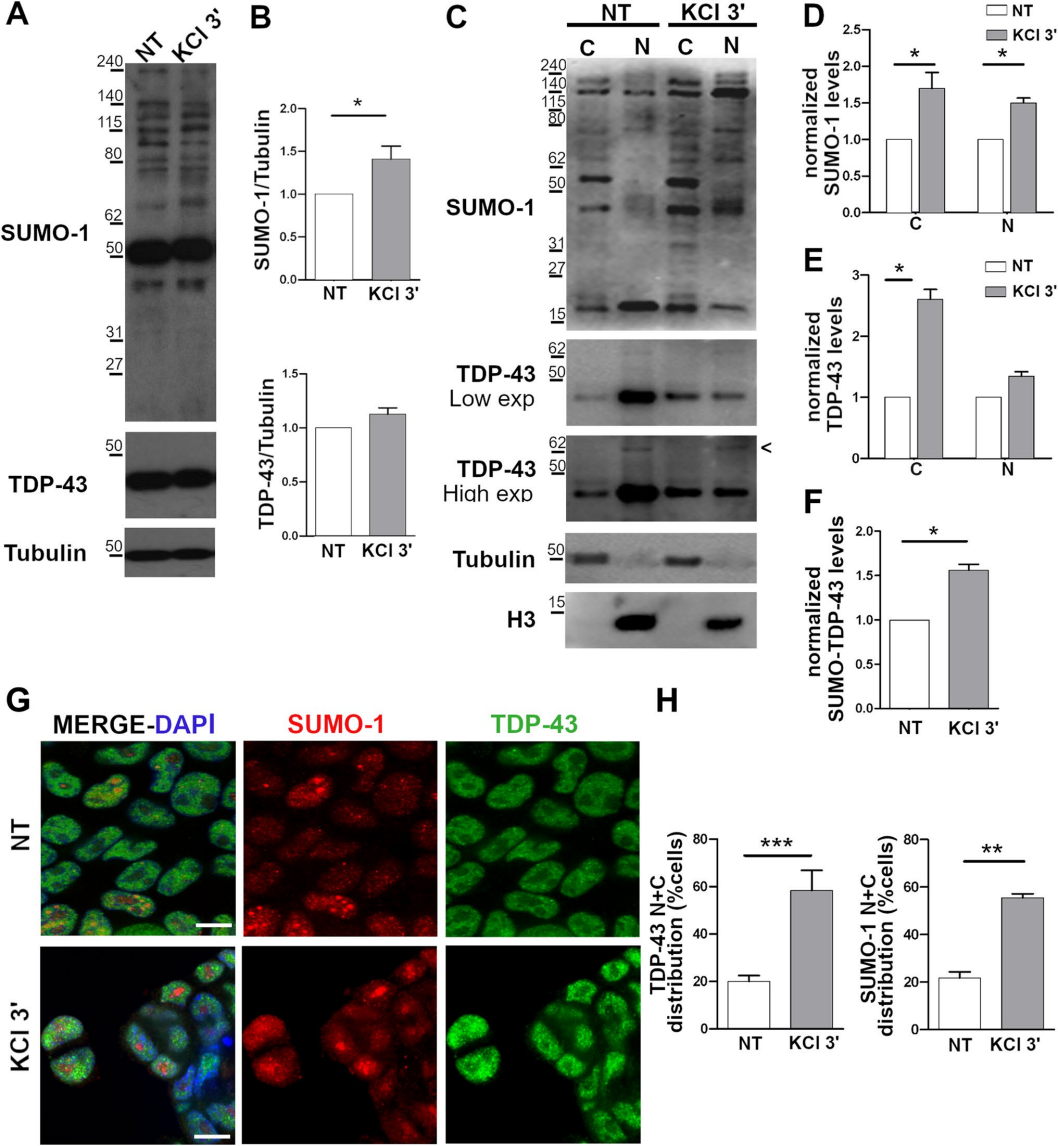
TDP-43 nucleocytoplasmic distribution upon positive modulation of SUMOylation by KCl treatment. (**a**) Representative WB images and (**b**) densitometric analyses of total SUMO-1 and TDP-43 protein levels in untreated (NT) and in KCl-treated (60 mM, 3 min) SK-N-BE cells. α-Tubulin was used for data normalization (mean ± s.e.m; *n* = 5 independent experiments; Unpaired t-test; *p <  0.05). (**c**) Representative WB images and (**d**) densitometric analyses of SUMO-1 protein levels in the nuclear (N) and cytoplasmic (C) fractions upon KCl treatment. An equal amount of lysates (40  g) was loaded for the two fractions. α-Tubulin and histone H3 were used as loading controls of the cytoplasmic and nuclear fractions, respectively (mean ± s.e.m; *n* = 5 independent experiments; Two-way ANOVA and Tukey post-test; *p <  0.05). *Arrowhead*, SUMOylated TDP-43 protein. Densitometric analysis of (**e**) TDP-43 protein levels in the nuclear (N) and cytoplasmic (C) fractions (mean ± s.e.m; *n* = 5 independent experiments; Two-way ANOVA and Tukey post-test; *p <  0.05) and (**f**) SUMOylated TDP-43 protein in the nuclear fraction (mean ± s.e.m; *n* = 5 independent experiments; Unpaired t-test; *p <  0.05). (**g**) IF images of endogenous TDP-43 (green) and SUMO-1 (red) in SK-N-BE cells treated with KCl. DAPI was used for nuclear staining. Scale bar, 10 µm. (**h**) Statistical analyses of the nuclear and cytoplasmic (N + C) distribution of SUMO-1 and TDP-43 proteins (mean ± s.e.m.; *n* = 4 independent experiments; at least 100 cells analysed/condition; Unpaired t-Test; **p <  0.01; ***p <  0.001)

To further study the effect of SUMOylation on TDP-43 trafficking, SK-NB-E cells were exposed to KCl stimulus (60 mM for 3 min), which is reported to up-regulate protein SUMOylation [57]. We observed a significant increase (1.4 ×) of total protein SUMOylation compared to untreated cells, while TDP-43 content was unchanged (Fig. 7a,b). By sub-cellular fractionation, we observed that KCl treatment induced a significant increase of total protein SUMOylation both in the nucleus (1.7 ×) and in the cytoplasm (1.5 ×) (Fig. 7c,d). KCl treatment also caused an increase of the amount of cytoplasmic TDP-43 (2.6 ×) (Fig. 7e) as well as of the SUMOylated TDP-43 form in the nucleus (1.6 ×) (Fig. 7f), although total TDP-43 protein levels did not significantly change (Fig. 7a,b,c). This result can be explained by the fact that both cytoplasmic TDP-43 and SUMO-TDP-43 represent only a minor fraction of total TDP-43 which is mostly nuclear and whose content may not be influenced significantly by such small variations.

By image analysis of TDP-43 sub-cellular distribution, we observed that KCl treatment induced a significant increase of the number of cells showing both nuclear and cytoplasmic localization of TDP-43 from 20.0% in physiological condition to 58.2% after exposure to KCl (Fig. 7g,h). No cells with a complete mislocalization of TDP-43 in the cytoplasm were observed. In line with these results, also the proportion of cells showing a nuclear and cytoplasmic localization of SUMO-1 increased from 21.7% to 55.5% upon KCl treatment (Fig. 7h).

### TDP-43 Pathological Aggregation After Treatment with the deSUMOylating TS-1 Peptide

**Fig. 8:**
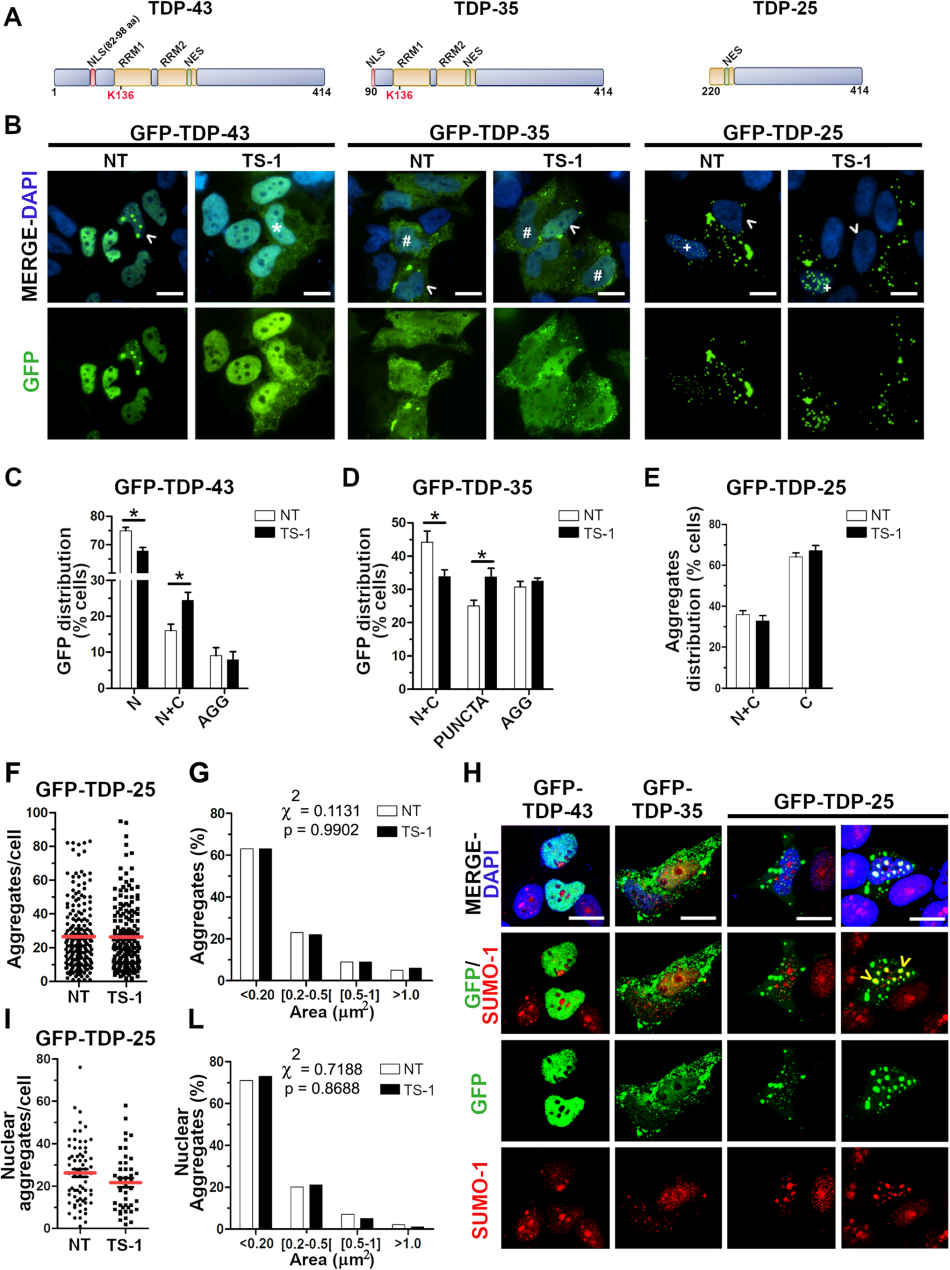
TDP-43 protein aggregation upon treatment with the deSUMOylating peptide TS-1. (**a**) Schematic representation of the TDP-43 full-length protein and the C-term fragments TDP-35 and TDP-25. The RNA-binding domains (RRM1, RRM2), the nuclear localization (NLS) and export (NES) signals and lysine 136 are highlighted. (**b**) IF images showing the GFP-TDP-43, GFP-TDP-35 and GFP-TDP-25 sub-cellular distribution in untreated (NT) and TS-1-treated (5  M, 4 h) SK-N-BE cells. Nuclei were stained with DAPI. Scale bar, 10 µm (*Asterisk*, cell with TDP-43 nuclear and diffused cytoplasmic distribution; *#*, cell with TDP-43 nuclear and a dotted cytoplasmic distribution (puncta); *arrowhead*, cell with nuclear or cytoplasmic TDP-43 aggregates; + , cell with cytoplasmic and nuclear aggregates). Statistical analyses of the sub-cellular distribution of GFP-TDP-43 (**c**) and GFP-TDP-35 (**d**) proteins upon TS-1 treatment (N, nuclear; N + C, nuclear and cytoplasmic; AGG, aggregates) (mean ± s.e.m; n ≥ 3 independent experiments; at least 80 cells analysed/condition; Two-way ANOVA and Bonferroni post-test; *p <  0.05). (**e**) Statistical analyses of GFP-TDP-25 aggregates distribution in the nucleus and the cytoplasm (N + C) and in the cytoplasm only (C) upon TS-1 treatment (mean ± s.e.m; *n* = 3 independent experiments; at least 80 cells analysed/condition; Two-way ANOVA and Bonferroni post-test). Quantitative analyses of GFP-TDP-25 aggregates number per cell (**f**) and size (**g**) (mean ± s.e.m; *n* = 4 independent experiments; at least 80 cells analysed/condition; Unpaired t-test in (**f**) and Chi-square test in (**g**)). (**h**) IF images showing GFP-TDP-43, GFP-TDP-35 and GFP-TDP25 proteins and the endogenous SUMO-1 (red) in SK-N-BE cells. DAPI was used for nuclei staining. Scale bar, 10 µm (*Arrowhead*, SUMO-1 and GFP-TDP-25 nuclear colocalization). Quantitative analyses of the number (**i**) and size (**l**) of GFP-TDP-25 aggregates specifically present in the nucleus, performed as in (**f**, **g**)

As the de-SUMOylating TS-1 peptide was shown to promote TDP-43 mislocalization in the cytoplasm and the process of TDP-43 aggregation is still poorly understood, we investigated the effect of TS-1 also on the distribution of the aggregation-prone TDP-43 C-terminal fragments (35KDa and 25KDa) (Fig. 8a). We first evaluated the sub-cellular localization of the full-length GFP-TDP-43 protein, which was mainly distributed in the nucleus (74.9% of SK-N-BE cells) with 16% of transfected cells showing a diffused localization of the exogenous protein also in the cytoplasm and 9.1% of cells showing GFP-positive nuclear or cytoplasmic aggregates (Fig. 8b,c). After 4-h-treatment with the TS-1 peptide (5 μM), we observed a significant decrease of the proportion of cells (67.7%) with only nuclear GFP-TDP-43 distribution and a significant increase of cells (24%) with also a cytoplasmic GFP-TDP-43 localization (Fig. 8b,c). Conversely, TS-1 treatment induced no changes in the distribution of GFP-positive aggregates (7.9% of cells) (Fig. 8c).

We then quantified the sub-cellular distribution of the GFP-TDP-35 protein, which lacks the first 89 N-terminal residues corresponding to the folded N-terminal domain [58], but includes part of the nuclear localization signal (NLS, 82–98 aa) and Lys136 (Fig. 8a). In contrast to GFP-TDP-43, GFP-TDP-35 was always diffused both in the nucleus and cytoplasm of 44.2% of transfected cells or showed a cytoplasmic dotted distribution (puncta) in 25.1% of GFP-positive cells and cytoplasmic aggregates in 30.7% of cells (Fig. 8b,d). Upon TS-1 treatment, the amount of cells with a nuclear and cytoplasmic distribution decreased to 33.8% and the percentage of cells with cytoplasmic GFP-TDP-35-positive puncta increased significantly to 33.7%, while the percentage of cells with cytoplasmic aggregates remained unchanged (32.4%) (Fig. 8d).

When we analysed the GFP-TDP-25 C-terminal fragment, deleted of the NLS, RRM1 and part of the RRM2 domain (Fig. 8a), it mainly formed aggregates, as previously reported [37], which were mostly distributed in the cytoplasm only (64.1% of transfected cells), but also both in the cytoplasm and in the nucleus (35.9% of GFP-positive cells) (Fig. 8b,e). TS-1 treatment did not change the sub-cellular distribution of GFP-TDP-25 aggregates in the two compartments (67.2% cells with cytoplasmic-only aggregates and 32.8% cells with cytoplasmic and nuclear aggregates) (Fig. 8e). To evaluate any possible effect of TS-1 treatment also on the formation of GFP-TDP-25 aggregates, we conducted a quantitative analysis of the number and size (area) of aggregates. We observed no differences either in the number of aggregates per cell (Fig. 8f) or in their size arbitrarily assigned to four different categories (< 0.2 µm^2^, [0.2–0.5[µm^2^, [0.5–1] µm^2^, > 1 µm^2^) after cell exposure to the TS-1 peptide (Fig. 8g).

Finally, we investigated if GFP-TDP-35 and GFP-TDP-25 colocalized with the endogenous SUMO-1 protein. By IF analysis, we observed that SUMO-1 largely colocalized with the nuclear GFP-TDP-25 aggregates, while GFP-TDP-43 and GFP-TDP-35 proteins or the cytoplasmic GFP-TDP-25 aggregates did not show any SUMO-1 colocalization (Fig. 8h). Given these results, we reconsidered our previous quantitative analysis on GFP-TDP-25-transfected cells by filtering our data specifically for the nuclear-only GFP-TDP-25 aggregates. Nonetheless, also in this analysis, we found that neither the number (26.1 vs 21.6 per cell) (Fig. 8i) nor the size (Fig. 8l) of nuclear GFP-TDP-25 aggregates changed upon TS-1 treatment.

## Discussion

PTMs regulate protein activity and interaction with their partners and contribute to finely tune a variety of cellular processes in a spatio-temporal manner. Among the PTMs described for TDP-43, including C-terminal cleavage, phosphorylation, ubiquitination and acetylation, SUMOylation has been only recently investigated [5, 59]. TDP-43 was reported to be modified by SUMO-1 in murine tissues and human experimental TDP-43 cell models [23, 24] and preliminary data already indicated that cytotoxic stress in HeLa cells was able to up-regulate protein SUMOylation, including that of TDP-43 [21].

In this work, we further extended the study of the functional link between SUMOylation and TDP-43 protein activity and trafficking. We first confirmed that human TDP-43 is SUMOylated in both neuronal and in non-neuronal cells and demonstrated that this PTM is limited to a small fraction of nuclear TDP-43. Indeed, similarly to other PTM, SUMOylation is a very dynamic and reversible process, which usually modifies a small proportion of the target protein to exert its regulatory function [60]. Importantly, we also showed that SUMO-1 can bind covalently and non-covalently in the N-terminal RRM1 domain, which is essential for TDP-43 RNA-binding activity, likely occurring at the predicted Lys 136 residue and at SIM3 106–110 aminoacidic sequence. While covalent SUMO-binding may primarily regulate the target protein activity, the non-covalent SUMO binding is supposed to favour the interaction with other proteins, similarly containing SIM motifs, and to act as a scaffold for the formation of multiprotein complexes. Compartmentalization of proteins in large ribonucleoprotein complexes is particularly important for the spatial organization and activity of splicing factors within the spliceosome in the nucleus [11]. Therefore, we speculate that TDP-43 may be spatially organized within the nucleus by SUMO scaffold proteins and that its splicing activity may be also partially modulated by SUMOylation at its RRM1 domain, as already proven for other splicing factors [9].

Supported by previous crystallographic studies [49, 50], our MD simulations indeed indicated that the putative SUMOylation site, the Lys 136 within the RRM1, is directly involved in mediating RNA target binding, and that this residue is also exposed and likely accessible to SUMO protein conjugation. When we investigated the impact of SUMOylation on TDP-43 splicing activity, we first found that the TDP-43 K136R mutant, whose folding and structure are similar to the wild-type protein, maintained the RNA-binding activity towards its targets although it was slightly decreased compared to the wild-type protein and to the mutant TDP-43 harbouring ALS-associated mutations in the C-terminal domain. While the decreased binding affinity towards its RNA targets seemed to account for the diminished exon skipping activity observed, on the other hand this did not influence the exon inclusion activity of TDP-43 K136R, which remained similar to the wild-type protein. Moreover, we proved that this different behaviour did not depend on the nature of the consensus binding sequence in the target RNA. These findings therefore suggest that, regardless of the RNA-binding capacity of the SUMO-mutant TDP-43 protein, TDP-43 splicing activity is specifically modulated by SUMOylation depending on the type of splicing event regulated, being exon inclusion preserved and exon skipping reduced.

Our results also show that TDP-43 recruitment into cytoplasmic SG upon induction of oxidative stress is completely impaired when the Lys 136 in the RRM1 domain is modified. We previously proved that the integrity of RRM1 and the C-terminal region spanning aminoacidic residues 216–315 are both needed for TDP-43 recruitment into SG [30] and, recently, the localization of TDP-43 into SG was further shown to be RNA-dependent [61]. However, also in this case, although the reduced, but not total loss of RNA-binding of the mutant protein may account for this observation, we can’t completely exclude that also SUMOylation of TDP-43 may play a role in regulating response to stress and recruitment of TDP-43 into SG. Altogether our results show that SUMOylation of TDP-43 in the nucleus may have an impact on the physiological activities of the protein because it is able to modify in part its exon skipping activity and RNA-binding capacity as well as its localization in SG in response to stress.

The understanding of the molecular mechanisms regulating TDP-43 trafficking and localization is pivotal to account for the cytoplasmic mislocalization and pathological aggregation of the protein observed in ALS/FTD brains. Several lines of evidence now suggest that defects not only in SG dynamics, but also in NCT may represent the initial steps of TDP-43 pathological deposition [62, 63]. When we focused on the possible effects of SUMOylation on TDP-43 nucleocytoplasmic trafficking, we found that modulation of the SUMOylation pathway impacted on TDP-43 sub-cellular localization, probably acting indirectly on the NCT system, which largely depends on RanGAP1 protein SUMOylation and its subsequent translocation to the nuclear membrane. By both over-expression of the de-SUMOylating enzyme SENP1 and treatment with the cell-permeable peptide TS-1, we showed that the cytoplasmic localization of both the exogenous and the endogenous TDP-43 significantly increased, as expected by a reduced activity of the RanGAP1 protein and, consequently, by a diminished import of TDP-43 into the nucleus. However, our results demonstrated that SUMOylation of TDP-43 protein itself is also important to regulate its nucleocytoplasmic shuttling because the SUMO-mutant TDP-43 K136R was less efficiently distributed in the cytoplasm upon induced deSUMOylation compared to the wild-type protein.

We further showed that this effect of SUMOylation on TDP-43 trafficking was specific by studying the NCT of two other splicing factors. In fact, while hnRNPA2/B1 was not predicted to be SUMOylated and its sub-cellular distribution was not influenced by SUMO1 or SENP1 overexpression, NOVA1 contains a putative lysine target and its NCT trafficking changed upon modulation of SUMOylation, although differently from TDP-43. This suggests the importance of the SUMO lysine targets and a more complex interplay of SUMOylation with other regulatory networks. Our observation that TDP-43 SUMO-modification regulates its sub-cellular distribution is supported also by a recent paper in which the mutant GFP-TDP-43 K136R formed less aggregates in the cytoplasm compared to the wild-type protein [24].

In the context of modulation of SUMOylation and NCT, we used an additional experimental paradigm to modulate this PMT, such as KCl stimulus, which is able to increase total protein SUMOylation [31]. We found that also KCl treatment induced SUMOylation of TDP-43 in the nuclear compartment and promoted, in parallel, its cytoplasmic localization. Modulation of SUMO PTM has the intrinsic limit to possibly impact on multiple pathways and protein targets, but the combined use of SUMO-resistant TDP-43 and different SUMOylation modulating factors (over-expression of SUMO-1, UBC9 and SENP1, treatments with TS-1 peptide and KCl) allowed us to better support our findings.

Since a negative modulation of SUMOylation by the cell-permeable peptide TS-1 induced a redistribution of TDP-43 in the cytoplasm, we further investigated if this had also an impact on the sub-cellular localization and aggregation of the pathological C-terminal TDP-43 fragments, p35 and p25, detected in ALS/FTD post-mortem brains. We found that the number of cells presenting with p35 protein diffused in the cytoplasm increased upon TS-1 treatment together with the proportion of cells showing small aggregates-like puncta. These data suggest that a negative modulation of SUMOylation similarly favoured the cytoplasmic localization of this truncated TDP-43 protein, which still contains the putative lysine 136, and in parallel also the formation of small aggregates, but had no effect on the already formed cytoplasmic aggregates. When we analysed the p25 fragment which, upon over-expression, forms exclusively aggregates both in the nucleus and in the cytoplasm, we observed no effect of TS-1 peptide on these truncated TDP-43 species although p25 aggregates seemed to colocalize with endogenous SUMO-1 specifically in the nucleus.

In the context of ALS/FTD diseases, the ageing process and the increase of oxidative stress occurring in post-mitotic neurons are known to dysregulate protein SUMOylation and NCT, possibly favouring an altered TDP-43 localization in the cytoplasm as an initial trigger of the protein aggregation process [64, 65]. The link between an altered TDP-43 trafficking and its cytoplasmic aggregation is also reinforced by the fact that the forming cytoplasmic aggregates interfere with the NCT in a feed-forward manner [65]. Therefore, our findings, by showing that TDP-43 sub-cellular localization in the cytoplasm may be regulated also by SUMOylation, help uncover all the molecular mechanisms causing TDP-43 cytoplasmic mislocalization as the initial event for its pathological aggregation in ALS/FTD and might be used to better define possible druggable targets. However, since TDP-43 undergoes different PTMs, including ubiquitination and acetylation, involving other lysine residues adjacent to the SUMO target Lys 136, like Lys 145 [5], the complex and possibly competitive interplay among SUMOylation and these PTMs in regulating TDP-43 function, localization and aggregation certainly deserves future exploration to fully understand such integrated regulatory networks in physiological as well as in pathological conditions.

## Supplementary Information

Below is the link to the electronic supplementary material.

Supplementary file1 (751 KB)

## Data Availability

The datasets generated during and/or analysed during the current study are available from the corresponding author on reasonable request. The summary statistics is available in Supplementary data.
