# Supplementary material for: SUMOylation Regulates TDP-43 Splicing Activity and Nucleocytoplasmic Distribution
Source: Mol Neurobiol. 2021 Aug 14;58(11):5682–702. doi: 10.1007/s12035-021-02505-8 (PMC8599232; doi:10.1007/s12035-021-02505-8)
Supplement: Supplementary file 1 — Supplementary file1 (751 KB) [file 12035_2021_2505_MOESM1_ESM.pdf]

SUPPLEMENTARY DATA

Supplementary Figure 1

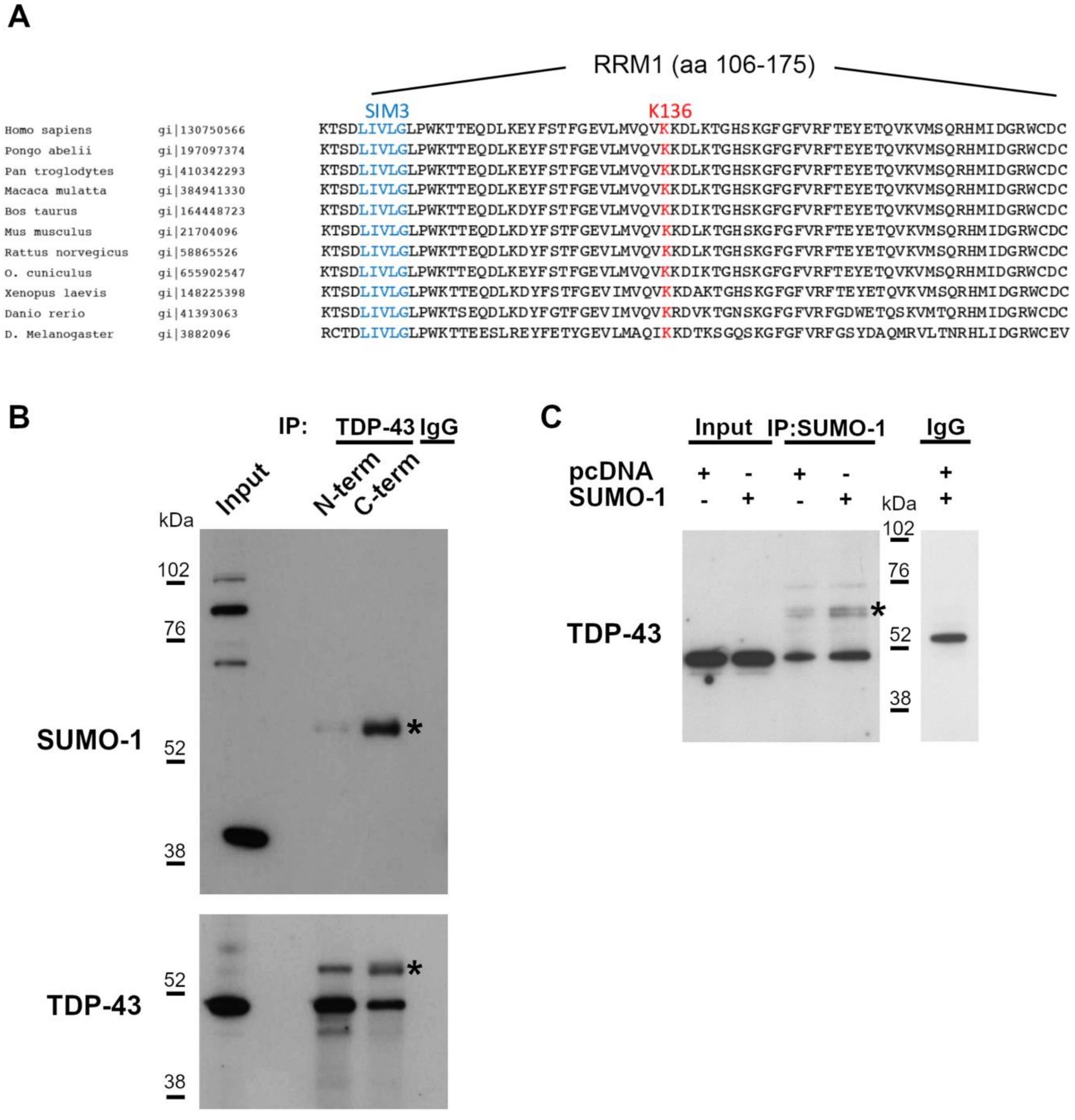

TDP-43 SUMOylation in HEK 293T cells. A) Phylogenetic analysis of the TDP-43 RRM1 sequence containing both the Lys 136 and the SIM3, as predicted by the *in silico* analyses. **B)** Representative WB images of immunoprecipitation (IP) assay on HEK 293T cell lysates treated with NEM to preserve protein SUMOylation. Anti-TDP-43 antibodies recognizing the N-term or C-term domain were used for IP. IgG antibody was used as negative control. **C)** Representative WB image of IP assay on HEK 293T cell lysates transfected with SUMO-1 or mock (pcDNA) constructs as indicated. Anti-SUMO-1 and IgG antibodies were used for IP. *Asterisk*, SUMOylated TDP-43 protein.

Supplementary Figure 2

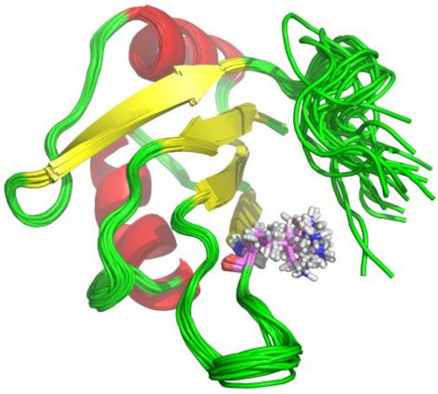

Conformational flexibility of Lys 136. The 20-lowest energy conformers making the NMR ensemble of TDP-43 RRM1 (PDB ID: 2cqg) is shown in red (helices), yellow (strands) and green (loops and disordered segments). K136 is depicted in stick representation (violet), highlighting that it both is not tied down to a specific conformation and remains flexible.

Supplementary Figure 3

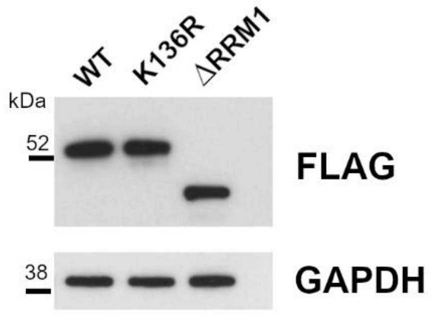

Expression of exogenous TDP-43 proteins upon transfection of HEK 293T cells. WB images showing the expression of the Flag-TDP-43 WT, K136R and ΔRRM1 proteins in HEK 293T cells after transfection. These protein lysates were used in the UV-CLIP assays showed in Figure 2.

Supplementary Figure 4

A

SUMO prediction site (NOVA-1)			
Lysine	JASSA	SUMO-plot	GPS-SUMO
59	no	low	medium
100	no	high	low
162	no	high	medium
198	no	high	low
219	no	high	high
453	no	high	low
474	low	low	medium
519	no	low	high

SIM prediction motif (NOVA-1)			
aa position	SIM sequence	JASSA	GPS-SUMO
105-109	...QTIVQLQKE...	medium	medium
132-136	...ERVCLIQGT..	no	medium
166-170	...EPVSILQPQ...	medium	medium
185-189	...AKLIVPNST...	no	medium
194-198	...AGLIIGKGG...	no	medium
229-233	...ERVVTISGE...	no	high
437-441	...KDVVEIAVP...	medium	high

B

SUMO prediction site (hnRNPA2B1)			
Lysine	JASSA	SUMO-plot	GPS-SUMO
110	no	no	medium

SIM prediction motif (hnRNPA2B1)			
aa position	SIM sequence	JASSA	GPS-SUMO
60-64	...SVLDRVLQE...	no	medium

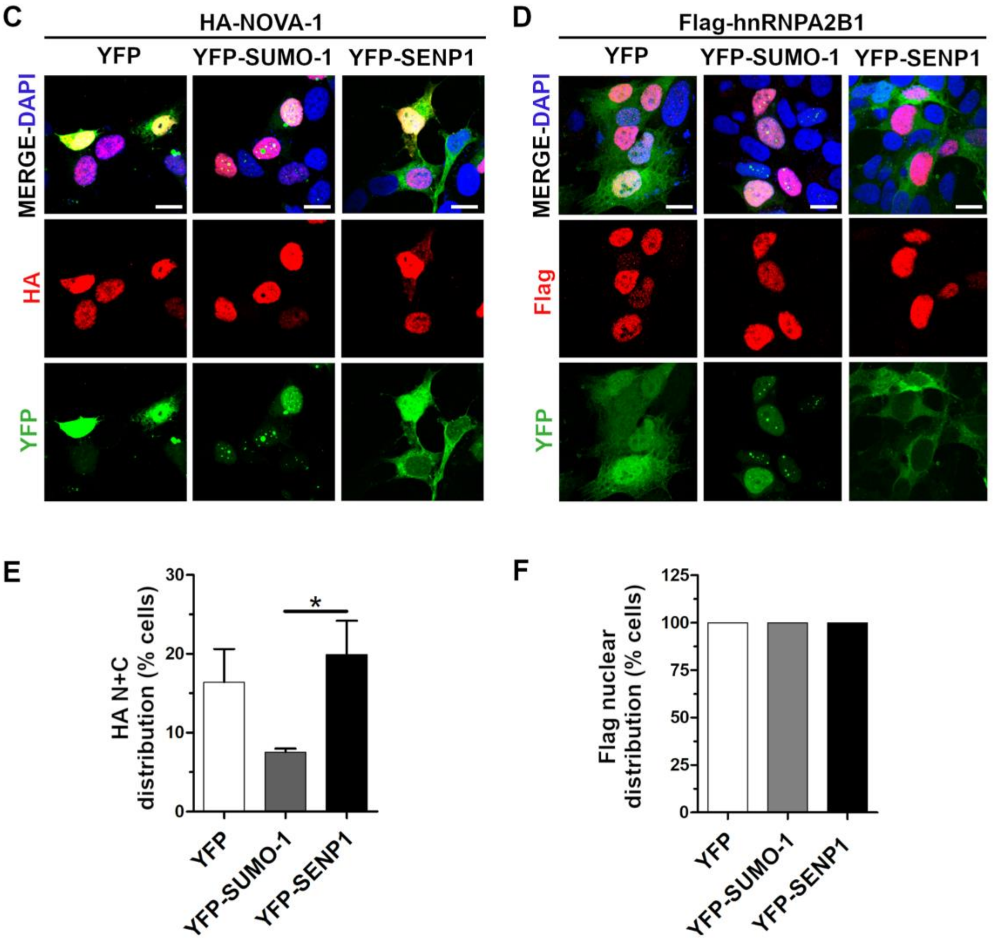

Modulation of SUMOylation and nucleo-cytoplasmic distribution of NOVA-1 and hnRNPA2B1. A-B) Prediction analyses of the putative SUMOylation sites (*left tables*) and SIM (*right tables*) of NOVA-1 **(A)** and hnRNPA2B1 **(B)** by using the indicated bioinformatic tools. **C,D)** Immunofluorescence images showing the sub-cellular distribution of HA-NOVA-1 **(C)** and Flag-hnRNPA2B1 **(D)** proteins in SK-N-BE cells upon YFP-SUMO-1 and YFP-SENP1 overexpression. DAPI was used for nuclei staining. Scale bar 10µm. **E,F)** Statistical analyses of both nuclear and cytoplasmic (N+C) distribution of HA-NOVA-1 **(E)** and of the nuclear distribution of Flag-hnRNPA2B1 **(F)** upon mock (YFP), YFP-SUMO-1 and YFP-SENP1 transfection (mean ± s.e.m.; n ≥3 independent experiments; at least 100 cells analysed per condition; One-way ANOVA and Tukey post hoc test; *p <0.05).

Supplementary Table 1.

List of primary and secondary antibodies used in Immunoprecipitation (IP), Immunofluorescence (IF) and Western Blot (WB) assays. Dilution conditions for WB and IF are indicated.

Antibody	Source	Assay
TDP-43 N-ter (1:1000/1:500)	Protein Tech_10782-2-AP	WB/IF/IP
TDP-43 C-ter	Protein Tech_12892-1-AP	IP
SUMO-1 (1:1000)	Santa Cruz_sc-5308	WB
SUMO-1 (1:50)	Cell Signaling_#4940	IF/IP
FLAG-Peroxidase (HRP) (1:1000)	Sigma-Aldrich_A8592	WB
Flag (1:2000)	Sigma-Aldrich_F1804	IF/IP
GFP (1:1000)	Protein Tech_50430-2-AP	WB
α Tubulin (1:500)	Santa Cruz_sc-8035	WB
H3 (1:1000)	Cell Signaling_#14269	WB
GAPDH (1:1000)	Santa Cruz_sc-47724	WB
TIAR (1:300)	Cell Signaling_#8509	IF
HA (1:200)	Sigma aldrich_H6908	IF
IgG mouse	Santa Cruz_sc-2025	IP
IgG rabbit	Santa Cruz_sc-2027	IP
Goat anti-mouse IgG-HRP (1:10000)	Santa Cruz_sc2005	WB
Goat anti-rabbit IgG-HRP (1:10000)	Santa Cruz_sc2004	WB
VeriBlot for IP Detection Reagent-HRP (1:1000)	Abcam_ab131366	WB
Goat anti-rabbit AlexaFluor 488 (1:500)	Thermo Fisher_#A-11034	IF
Goat anti-rabbit AlexaFluor 555 (1:500)	Thermo Fisher_#A-21430	IF
Goat anti-mouse AlexaFluor 488 (1:500)	Thermo Fisher_#A-11017	IF
Goat anti-mouse AlexaFluor 555 (1:500)	Thermo Fisher_#A-21422	IF

Supplementary Table 2. List of primers used for RT-PCR in splicing assays.

Gene	Foward primer	Reverse primer	Ref
GAPDH	TCCCCACTGCCAACGTGTCAGTG	ACCCTGTTGCTGTAGCCAAATTCG	Colombrita et al, 2015
MADD	GACCTGAATTGGGTGGCGAGTTCCCT	CATTGGTGTCTTGTACTTGTGGCTC	De Conti et al, 2015
STAG2	GTATGTTTACTTGGAAAAGTTCATG	TGATTCATCCATAATTGAAGCTGGA	De Conti et al, 2015
POLDIP3	GCTTAATGCCAGACCGGGAGTTG	TCATCTTCATCCAGGTCATATAAATT	Colombrita et al, 2015

Supplementary Table 3. Summary of statistical analyses.
DF, degree of freedom.

	Number of experiments	Mean±SD	Statistical test; DF	Post *hoc* test
**Figure 3**				
3A	siCTRL: 3<br>siTDP-43: 3<br>siTDP-43+Flag-TDP-43 WT: 3<br>siTDP-43+Flag-TDP-43 K136R: 3	8.43%±1.84%<br>3.23%±2.75%<br>63.98%±7.5%<br>27.50%±0.47%	one way-ANOVA;<br>DF: 11	Tukey
3B	siCTRL: 4<br>siTDP-43: 4<br>siTDP-43+Flag-TDP-43 WT: 4<br>siTDP-43+Flag-TDP-43 K136R: 4	64.41%±6.97%<br>24.70%±10.01%<br>91.01%±3.08%<br>64.37%±5.15%	one way-ANOVA;<br>DF: 15	Tukey
3C	siCTRL: 3<br>siTDP-43: 3<br>siTDP-43+Flag-TDP-43 WT: 3<br>siTDP-43+Flag-TDP-43 K136R: 3	3.65%±1.32%<br>2.34%±1.12%<br>12.47%±4.9%<br>2.26%±0.84%	one way-ANOVA;<br>DF: 11	Tukey
3D	siCTRL: 6<br>siTDP-43: 6<br>siTDP-43+Flag-TDP-43 WT: 6<br>siTDP-43+Flag-TDP-43 K136R: 6	91.08%± 0.39%<br>89.67%±0.73%<br>93.88%±0.59%<br>93.77%±1.06%	one way-ANOVA;<br>DF: 23	Tukey
3F	siCTRL: 3<br>siTDP-43: 3<br>siTDP-43+Flag-TDP-43 WT: 3<br>siTDP-43+Flag-TDP-43 K136R: 3	92.44%±5.09%<br>69.55%±3.08%<br>79.93%±0.9%<br>79.6%±2.89%	one way-ANOVA;<br>DF: 11	Tukey
3G	siCTRL: 3<br>siTDP-43: 3<br>siTDP-43+Flag-TDP-43 WT: 3<br>siTDP-43+Flag-TDP-43 K136R: 3	81.73%±2.92%<br>63.52%±3.72%<br>74.13%±3.14%<br>65.74%±1.4%	one way-ANOVA;<br>DF: 11	Tukey
3H	siCTRL: 5<br>siTDP-43: 5<br>siTDP-43+Flag-TDP-43 WT: 5<br>siTDP-43+Flag-TDP-43 K136R: 5	77.98%±5.32%<br>29.82%±5.05%<br>42.52%±4.6%<br>47.7%±10.32%	one way-ANOVA;<br>DF: 19	Tukey
**Figure 5**				
5B	Flag-TDP-43 WT+YFP: 3<br>Flag-TDP-43 WT+YFP-SUMO-1: 3<br>Flag-TDP43 WT+YFP-SENP1: 3<br>Flag-TDP-43 K136R+YFP: 3<br>Flag-TDP-43 K136R+YFP-SUMO-1: 3<br>Flag-TDP-43 K136R+YFP-SENP1: 3	3.71%±0.93%<br>3.93%±0.97%<br>19.27%±2.77%<br>3.28%±0.59%<br>2.77%±0.74%<br>9.04%±0.74%	two way-ANOVA;<br>DF:29	Bonferroni
**Figure 6**				
6B_SUMO-1	NT: 5<br>TS-1: 5	0.42±0.058	Student's Unpaired *t*-<br>Test; DF:6	N/A
6B_TDP-43	NT: 5<br>TS-1: 5	0.74±0.07	Student's Unpaired *t*-<br>Test; DF:6	N/A
6D_SUMO-2	NT: 4<br>TS-1: 4	21.65%±2.7%<br>37.25%±1.49%	Student's Unpaired *t*-<br>Test; DF:6	N/A
6D_TDP-43	NT: 4<br>TS-1: 4	20%±1.25%<br>25.5%±0.64%	Student's Unpaired *t*-<br>Test; DF:6	N/A

	Number of experiments	Mean±SEM	Statistical test; DF	Post *hoc* test
**Figure 7**				
7B_SUMO-1	NT: 5<br>KCL 3': 5	1.41±0.15	Student's Unpaired *t*-Test; DF:4	N/A
7B_TDP-43	NT: 5<br>KCL 3': 5	1.126±0.058	Student's Unpaired *t*-Test; DF:8	N/A
7D_SUMO-1	C_KCL 3': 5<br>N_KCL 3': 5	1.69±0.21<br>1.5±0.06	two way-ANOVA; DF:5	Tukey
7E_TDP-43	C_KCL 3': 5<br>N_KCL 3': 5	2.6±0.161<br>1.34±0.074	two way-ANOVA; DF:5	Tukey
7E_SUMO-TDP-43	NT: 5<br>KCL 3': 5	1.56±0.065	Student's Unpaired *t*-Test; DF:6	N/A
7G_TDP-43	NT: 4<br>KCL 3': 4	20%±1.22%<br>58.25%±4.25%	Student's Unpaired *t*-Test; DF:6	N/A
7G_SUMO-1	NT: 4<br>KCL 3': 4	21.75%±2.59%<br>55.5%±2.59%	Student's Unpaired *t*-Test; DF:6	N/A
**Figure 8**				
8C	NT_N: 4<br>NT_N+C: 4<br>NT_AGG: 4<br>TS-1_N: 4<br>TS-1_N+C: 4<br>TS-1_AGG: 4	74,9%±1.22%<br>16.01%±1.84%<br>9.09%±2.21%<br>67.7%±1.37%<br>24.37%±2.28%<br>7.94%±2.22%	two way-ANOVA; DF: 23	Bonferroni
8D	NT_DIffuse: 3<br>NT_Puncta: 3<br>NT_AGG:3<br>TS-1_Diffuse:3<br>TS-1_Puncta: 3<br>TS-1_AGG: 3	44.21%±3.3%<br>25.05%±1.68%<br>30.73±1.64%<br>33.81%±2.05%<br>33.75%±2.54%<br>32.44%±0.96%	two way-ANOVA; DF: 17	Bonferroni
8E	NT_N: 4<br>NT_C: 4<br>TS-1_N: 4<br>TS-1_C: 4	35.89%±1.97%<br>64.12%±1.97%<br>32.85%±2.55%<br>67.16%±2.55%	two way-ANOVA; DF: 12	Bonferroni
8F_Aggregates	NT: 4<br>TS-1: 4	26.56±1.4<br>26.36±1.47	Student's Unpaired t-Test; DF: 364	N/A
8F_AGG Distribution	NT vs TS-1:<br><0.20 µm: 4<br>[0.2-0.5 µm[: 4<br>[0.5-1 µm]: 4<br>>1.0 µm: 4	NT vs TS-1:<br>63 vs 63<br>23 vs 22<br>9 vs 9<br>5 vs 5	Chi-square; DF:3	N/A
8H_Aggregates	NT: 4<br>TS-1: 4	26.13±1.78<br>21.63±2.13	Student's Unpaired t-Test; DF: 110	N/A
8H_AGG Distribution	NT vs TS-1:<br><0.20 µm: 4<br>[0.2-0.5 µm[: 4<br>[0.5-1 µm]: 4<br>>1.0 µm: 4	NT vs TS-1:<br>71 vs 73<br>20 vs 21<br>7 vs 5<br>2 vs 1	Chi-square; DF:3	N/A
**Supplementary Figure 4**				
4E	YFP: 4<br>YFP-SUMO-1: 4<br>YFP-SENP1: 4	16.4%±4.2%<br>7.53%±0.47%<br>19.9%±4.29%	one way-ANOVA; DF:11	Tukey
4F	YFP: 3<br>YFP-SUMO-1: 3<br>YFP-SENP1: 3	100%±0%<br>100%±0%<br>100%±0%	N/A	N/A
